# The Influence of Polyphosphoric Acid Dosage on the Physical, Rheological, Ageing, and Microstructural Characteristics of Bitumen

**DOI:** 10.3390/ma19214687

**Published:** 2026-11-03

**Authors:** Anwaar Hazoor Ansari, Gailan Ismat Safaeldeen, Raza Mehmood, Zafreen Elahi, Fauzan Mohd Jakarni, Syed Nasir Shah, Masood Ahmed

**Affiliations:** 1 Department of Civil Engineering, Faculty of Engineering, Balochistan University of Information Technology, Engineering, and Management Sciences (BUITEMS), Quetta 87300, Pakistan; zafrin.elahi@buitms.edu.pk (Z.E.); syed.nasir@buitms.edu.pk (S.N.S.); masood.ahmed2@buitms.edu.pk (M.A.); 2 Technical Engineering College–Kirkuk, Northern Technical University, Kirkuk 36001, Iraq; 3 Department of Civil Engineering, Faculty of Engineering, Universiti Putra Malaysia, Serdang 43400 UPM, Selangor, Malaysia; gs64715@student.upm.edu.my (R.M.); fauzan.mj@upm.edu.my (F.M.J.)

**Keywords:** polyphosphoric acid, bitumen, rheological properties, ageing, temperature susceptibility, microstructure

## Abstract

Polyphosphoric acid (PPA) is an effective chemical modifier for improving the performance characteristics of bitumen. This study investigated the influence of PPA dosage on the physical, rheological, ageing, and microstructural characteristics of 60/70 penetration grade bitumen. PPA with 115% H_3_PO_4_ equivalent concentration was incorporated at 0.5%, 1.0%, 1.5%, and 2.0% by weight of bitumen. The binders were evaluated through conventional physical tests, ageing indices, dynamic shear rheology, master curves, Black and Cole–Cole diagrams, field emission scanning electron microscopy (FESEM), and Fourier transform infrared spectroscopy (FTIR). Increasing PPA concentration reduced penetration from 62 to 24 dmm and increased the softening point from 46.5 to 68 °C, while viscosity at 135 °C increased from 0.38 to 1.83 Pa·s. The Penetration Index (PI) and Penetration Viscosity Number (PVN) results also indicated reduced temperature susceptibility. Rheological evaluation showed a pronounced response at approximately 1.0% PPA, where G*/sinδ increased from 1.01 to 12.35 kPa and the high temperature grade increased from Performance Grade (PG)64 to PG 82. Master curves showed a stronger PPA influence at lower reduced frequencies, while Black and Cole–Cole analyses indicated an increased relative elastic contribution. These rheological characteristics remained evident following Rolling Thin Film Over (RTFO) ageing, although the ageing response varied with PPA concentration and the evaluated property. FESEM showed homogeneous PPA incorporation without visible phase separation, while FTIR revealed progressive changes in the phosphate-related spectral region. Among the investigated dosages, 1.0% PPA provided the most balanced performance based on the physical properties, high temperature rheological response, short-term ageing behaviour, and microstructural characteristics evaluated in this study. However, further evaluation of low temperature and long-term ageing performance is required before establishing an overall optimum PPA dosage.

## 1. Introduction

Bitumen is a complex viscoelastic material whose mechanical response is strongly influenced by temperature, loading time, and loading frequency. At higher temperatures and prolonged loading conditions, the increased viscous response of bitumen can contribute to permanent deformation, whereas excessive stiffness at lower temperatures can adversely affect its ability to accommodate stresses and strains. In addition to temperature and loading conditions, bituminous binders undergo progressive ageing during asphalt production, transportation, construction, and subsequent service. Short-term ageing is primarily associated with volatilization and oxidation at higher temperatures, resulting in changes in binder composition, stiffness, viscosity, and overall rheological behaviour [1,2,3,4]. These changes can significantly influence pavement resistance to deformation and cracking and ultimately affect the durability and service life of asphalt pavements [1,2]. Consequently, improving the temperature susceptibility, rheological response, and ageing characteristics of conventional bitumen continues to receive considerable attention in pavement materials research.

Various modification approaches have been developed to enhance the performance of conventional bitumen, including polymeric modifiers, crumb rubber, chemical additives, and composite modification systems [5,6,7,8,9]. Among chemical modifiers, polyphosphoric acid, PPA, has received considerable attention because relatively small quantities can produce substantial changes in binder consistency and high temperature performance [5,6,7,8]. PPA consists of a distribution of phosphoric acid species with different degrees of condensation, and its modification mechanism differs from that of conventional polymeric modifiers [8,10]. The response of bitumen to PPA is also strongly dependent on the chemical composition and source of the base binder. Baumgardner et al. [5] demonstrated that PPA can affect different phases within bitumen depending on the characteristics of the original binder, indicating that its modification effect cannot be considered independent of base binder chemistry. Subsequent research has continued to demonstrate the effectiveness of PPA for improving high temperature binder performance, although its influence on other performance characteristics can vary considerably with binder composition, PPA characteristics, and modifier dosage [7,8].

The dosage of PPA is an important factor governing the resulting properties of modified bitumen. Previous studies have generally reported that increasing PPA content increases binder consistency, viscosity, temperature stability, and resistance to permanent deformation [4,8,10,11,12]. Jiang et al. [11] investigated PPA contents ranging from 0.5% to 2.5% and reported a progressive reduction in penetration together with increases in softening point, penetration index, viscosity, and resistance to nonrecoverable deformation. The influence of PPA is not necessarily proportional throughout the entire dosage range, however, and the magnitude of improvement can vary among different performance characteristics. High temperature flow behaviour has similarly been shown to depend strongly on PPA concentration and temperature, with increasing PPA content producing considerable changes in binder viscosity and flow characteristics [12]. Therefore, increasing the amount of PPA does not necessarily represent the most effective modification strategy, and an appropriate dosage should be established by considering the overall response of the modified binder rather than an individual performance indicator.

Beyond changes in conventional consistency, PPA substantially affects the viscoelastic characteristics of bitumen. Previous investigations have generally demonstrated increased stiffness and a more elastic response following PPA modification, particularly under conditions associated with high pavement temperatures and slow traffic loading [4,8,10,11,12,13]. These changes contribute to improved resistance to permanent deformation and are among the principal advantages associated with PPA modification. However, the magnitude of the rheological response depends on several factors, including modifier dosage, temperature, loading conditions, and the chemical characteristics of the base binder [4,5,11,12]. PPA can also alter the internal organisation and interactions among bituminous fractions, thereby affecting binder response over a broad range of loading and temperature conditions [3,9]. Consequently, the characterisation of PPA-modified bitumen requires consideration of its overall viscoelastic response rather than relying exclusively on changes in conventional consistency or a single performance parameter. Ageing represents an additional consideration because PPA modification and oxidative ageing can simultaneously increase binder stiffness. During short-term ageing, oxidation and the loss of volatile constituents alter the physicochemical characteristics of bitumen and generally increase its stiffness and viscosity [1,2,3]. Previous studies have demonstrated that PPA can influence the ageing response of bituminous binders, although the extent of this influence depends on PPA dosage, base binder composition, and the presence of other modifiers [4,14,15]. This interaction creates an important distinction between modification-induced stiffening and ageing-induced stiffening. An increase in stiffness following PPA modification may improve resistance to permanent deformation, whereas excessive stiffening resulting from ageing may negatively affect other aspects of binder performance. Therefore, understanding how PPA dosage influences the susceptibility of bitumen to short-term ageing is necessary for evaluating its overall effectiveness rather than considering only the properties of the unaged, modified binder.

The performance changes associated with PPA modification are closely related to interactions between the modifier and the chemical constituents of bitumen. Earlier mechanistic investigations indicated that PPA can modify associations within different binder fractions and that the nature of these interactions varies with base binder chemistry [5,6]. Recent studies have further demonstrated that PPA-induced changes in molecular and colloidal characteristics correspond with substantial changes in rheological behaviour [7,16]. This relationship between physicochemical characteristics and engineering performance is important because similar increases in macroscopic stiffness may arise from different structural mechanisms. Establishing whether progressive PPA modification produces corresponding changes in binder structure and whether these changes are consistent with observed performance trends can therefore contribute to a more complete understanding of the modification process. Although considerable research has established the beneficial influence of PPA on bitumen, particularly in terms of high temperature performance, several aspects require further integrated evaluation. Previous investigations have frequently focused on specific performance characteristics, particular ageing conditions, or PPA used as a component of composite modification systems [4,7,8,15,17]. Consequently, the relationship among progressive PPA dosage, temperature susceptibility, short-term ageing response, and the evolution of overall viscoelastic behaviour is not always evident when these characteristics are considered independently. Furthermore, relating changes in engineering performance to the corresponding physicochemical and morphological characteristics of the binder can provide additional insight into the mechanisms responsible for the observed response. An integrated evaluation of these aspects is therefore necessary to distinguish simple binder stiffening from broader changes in the performance and internal characteristics of PPA-modified bitumen.

Accordingly, the present study aims to comprehensively investigate the dosage dependent influence of 115% PPA on the physical, rheological, and short-term ageing characteristics of bitumen. Particular attention is given to determining how progressive PPA modification influences temperature susceptibility, ageing response, and viscoelastic behaviour over a broad range of temperature and loading conditions. The study further examines the relationship between the observed performance changes and the corresponding physicochemical and morphological characteristics of the modified binder. Through this integrated approach, the study seeks to provide a more comprehensive understanding of PPA modification and to identify a suitable PPA dosage considering the overall balance of binder performance.

## 2. Materials and Methods

**Figure 1:**
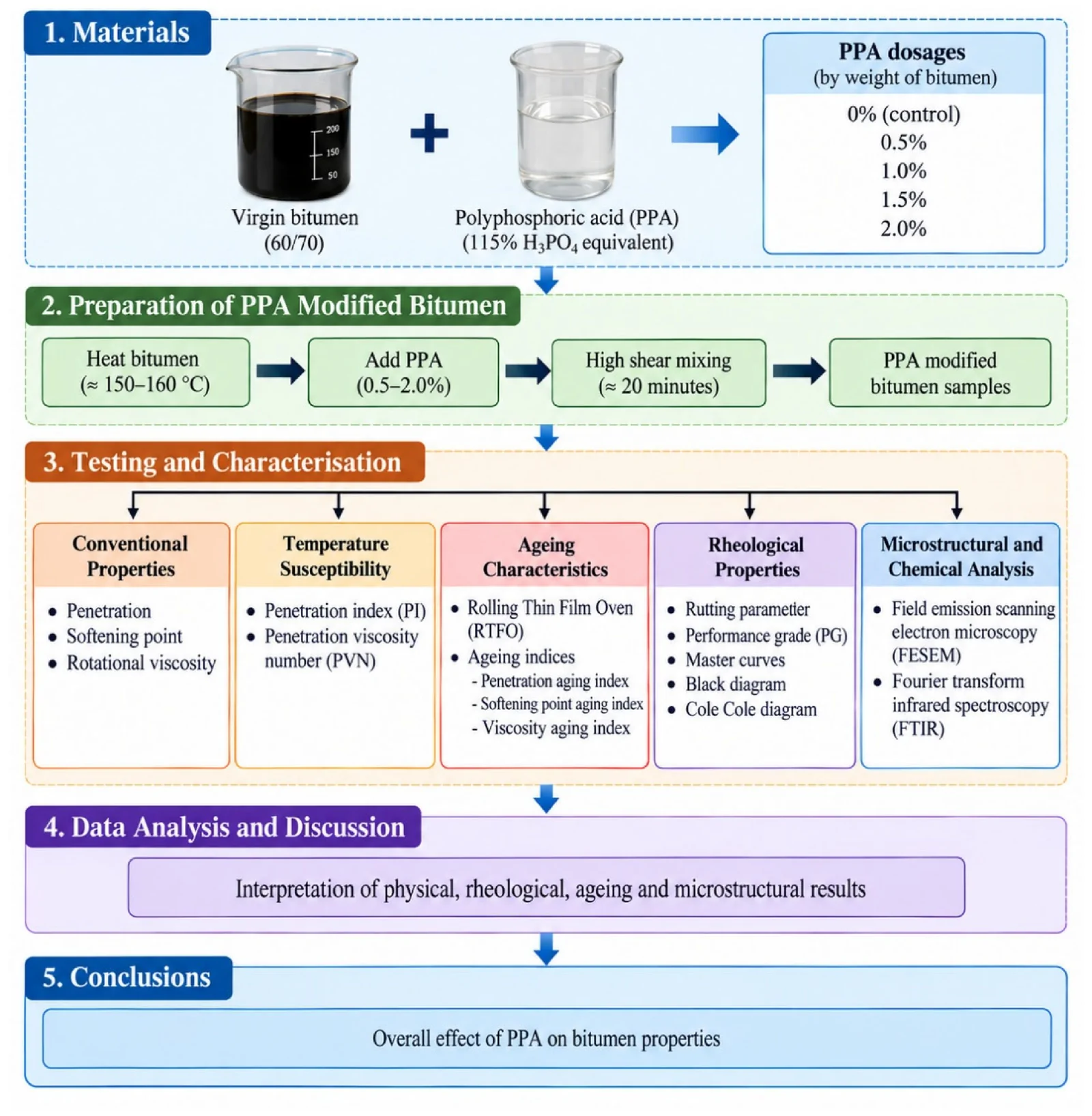
An experimental flow chart of the study.

This section describes the properties of the materials used; the procedures adopted for sample preparation; and the physical, rheological, and microstructural testing methods employed in the study, along with the relevant standards followed. Figure 1 presents a detailed flowchart illustrating the overall research methodology.

### 2.1. Materials

**Figure 2:**
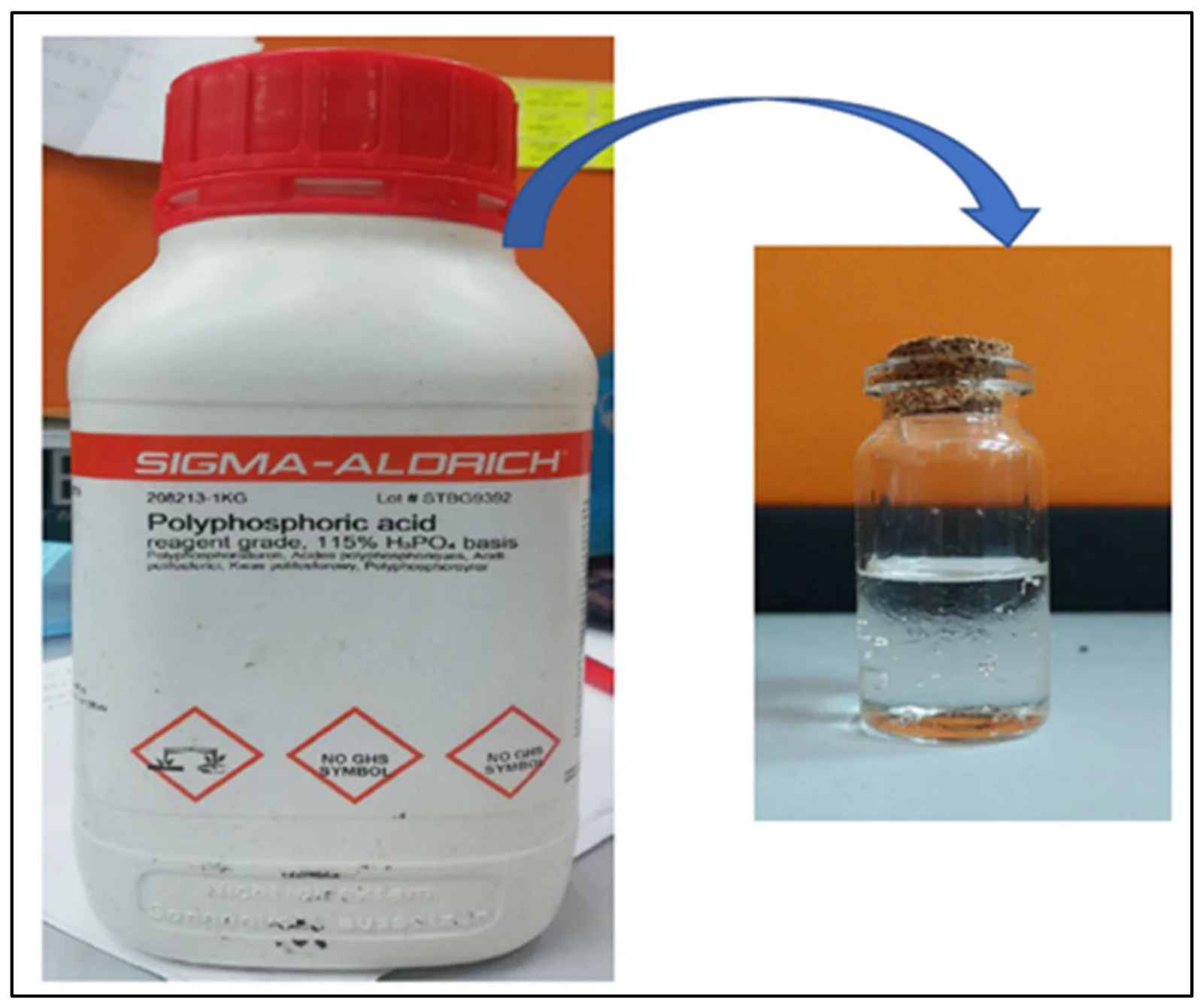
H_3_PO_4_ equivalent polyphosphoric acid used for bitumen modification in experimental programme.

Penetration grade 60/70 bitumen, supplied by KR Premix Holdings Sdn Bhd, Semenyih, Selangor Malaysia, was used as the base binder in this study. The selected bitumen contained no additional modifiers or additives, allowing the influence of polyphosphoric acid, PPA, to be evaluated independently. Polyphosphoric acid (PPA, reagent grade, 115% H_3_PO_4_ basis, Sigma Aldrich) was procured from Synergy Scientific Sdn. Bhd., Kuala Lumpur, Malaysia, and had a concentration equivalent to 115% H_3_PO_4_, as shown in Figure 2. The basic properties of the virgin bitumen and PPA are presented in Table 1.

### 2.2. Preparation of Modified Bitumen

The PPA-modified bitumens were prepared by incorporating PPA into the base bitumen at concentrations of 0.5%, 1.0%, 1.5%, and 2.0% by the weight of the bitumen, while the unmodified bitumen containing 0% PPA was used as the control. The base bitumen was initially heated to 150 °C to obtain sufficient fluidity for mixing. The required quantity of PPA was then gradually added to the heated bitumen and mixed using IKA RW 20 DZM mechanical overhead stirrer (IKA Werke GmbH & Co. KG, Staufen, Germany) for approximately 20 min. The gradual addition of PPA was adopted to facilitate uniform incorporation of the modifier throughout the bitumen. During preparation, the temperature was maintained at approximately 150 °C (±2 °C), and continuous mixing was carried out until a visually homogeneous binder was obtained. Care was taken during mixing to minimise unnecessary air entrainment. Following the completion of mixing, the prepared binders were allowed to settle sufficiently to facilitate the release of entrapped air bubbles before being transferred to storage containers. The prepared binders were subsequently stored in closed containers under a controlled laboratory environment to minimise contamination, moisture exposure, and unnecessary oxidative ageing prior to testing.

### 2.3. Conventional Bitumen Testing

The conventional properties of the control and PPA-modified bitumens were evaluated through penetration, softening point, and rotational viscosity tests. These tests were selected to assess changes in binder consistency and flow characteristics resulting from progressive PPA modification. All modified bitumens were tested under identical conditions to allow direct comparison with the control bitumen. The penetration test was conducted at 25 °C to determine binder consistency, while the softening point was determined using the ring and ball method to assess the response of the binder to increasing temperature. Rotational viscosity was measured at 135 °C and 165 °C to evaluate the flow characteristics of the binders at higher temperatures. The testing conditions and corresponding ASTM standards are summarised in Table 1. All measurements were performed using three specimens and expressed as mean ± standard deviation. The effect of PPA dosage was evaluated using one-way Analysis of Variance ANOVA followed by Tukey’s Honestly Significant Difference HSD, with statistical significance considered at *p* < 0.05. In addition to the measured conventional properties, the penetration index (PI) and penetration viscosity number (PVN) were calculated to assess the temperature susceptibility of the control and PPA-modified bitumens. PI was determined from the penetration and softening point values, whereas PVN was determined using the penetration and viscosity results.

### 2.4. Rheological Characterisation and Modelling

The rheological characteristics of the control and PPA-modified bitumens were evaluated using a Dynamic Shear Rheometer (DSR), HAAKE RheoStress 1 (Thermo Fisher Scientific, Karlsruhe, Germany)., in accordance with AASHTO T315. The DSR was used to determine the complex shear modulus (G*), and phase angle (δ), which represent the overall resistance to deformation and the relative viscous and elastic response of the bitumen, respectively. The rheological testing programme consisted of temperature sweep and frequency sweep tests conducted within the linear viscoelastic region of the binders. The temperature sweep test was conducted over the selected temperature range (40–82 °C) at a constant loading frequency of 1.59 Hz. The resulting G* and δ values were used to evaluate the temperature-dependent rheological response of the control and PPA-modified bitumens. The rutting parameter (G*/sin δ) was also determined to assess the resistance of the binders to permanent deformation at higher temperatures. The temperature sweep evaluation was performed for both the unaged and RTFO-aged binders, allowing the effect of PPA concentration and short-term ageing on the rheological response to be compared.

The frequency sweep test was performed over a frequency range of 0.1 to 13 Hz at 35–85 °C within the linear viscoelastic response of the binders. The variation in G* and δ with loading frequency and temperature was used to characterise the viscoelastic behaviour of the control and PPA-modified binders over a wider range of loading conditions. The frequency sweep data were subsequently used to construct Black space diagrams and Cole–Cole diagrams, providing additional assessment of the changes in rheological behaviour associated with progressive PPA modification. The G′ and G″ values used for the Cole–Cole diagrams were obtained directly from the DSR frequency sweep measurements. The Black space and Cole–Cole diagrams were constructed directly from the experimental rheological data.

### 2.5. Short-Term Ageing and Ageing Indices

The short term ageing of the control and PPA modified bitumens was performed using a rolling thin film oven (RTFO; S.E.M. (S.A.) Pty. Ltd., Magill, SA, Australia), in accordance with ASTM D2872. Following ageing, the binders were evaluated using the selected conventional and rheological tests to determine the influence of PPA concentration on ageing susceptibility. The changes in conventional properties due to RTFO ageing were further quantified using the penetration ageing index, softening point ageing index, and viscosity ageing index, based on the corresponding properties measured before and after ageing. These indices were used to compare the short-term ageing response of the control and PPA-modified bitumens.

### 2.6. Master Curve

The frequency sweep data were used to construct master curves for the control and PPA-modified bitumens based on the time temperature superposition principle. The tests were performed over a frequency range of 0.1 to 13 Hz at temperatures ranging from 35 °C to 85 °C, with an interval of 10 °C. A reference temperature of 65 °C was selected for master curve development. The experimental data obtained at different temperatures were shifted horizontally to the reference temperature to extend the rheological response over a broader frequency domain. The shift factors were determined using the Williams Landel Ferry (WLF), model, as expressed in Equation (1).

(1) $$\log\alpha_{T}=\frac{C_{1}\left(T-T_{0}\right)}{C_{2}+\left(T-T_{0}\right)}$$

where $\alpha_{T}$ is the shift factor, T is the testing temperature, $T_{0}$ is the reference temperature of 65 °C, and $C_{1}$ and $C_{2}$ are empirical constants.

Following determination of the shift factors, the complex shear modulus data were fitted using the sigmoidal rheological model, expressed as:

(2) $$\log|G^{*}|=\nu +\frac{\alpha }{1+e^{\left(\beta +\gamma \log f_{r}\right)}}$$

where ν represents the position of the lower asymptote, $f_{r}$ is the reduced frequency, α represents the difference between the lower and upper asymptotes, while β  and γ define the shape of the sigmoidal function between the asymptotes. The shifted data were fitted at the reference temperature of 65 °C to obtain smooth master curves for comparison of the rheological response of the control and PPA-modified bitumens.

### 2.7. Field Emission Scanning Electron Microscope (FESEM)

The surface morphology of the control and PPA-modified bitumens was examined using field emission scanning electron microscopy (FESEM) to investigate the morphological changes associated with PPA modification and short-term ageing. The analysis was performed using a Nova NanoSEM 230 field emission scanning electron microscope (FESEM; FEI Company, Brno, Czech Republic). Prior to imaging, a small quantity of each bitumen sample was prepared and coated with a thin layer of platinum to provide the electrical conductivity required for observation. The analysis was performed on both unaged and RTFO-aged binders to examine the changes in surface morphology resulting from ageing and to determine how PPA modification influenced these changes. Micrographs were obtained at selected magnifications for the control and different PPA concentrations. The morphological assessment focused primarily on changes in surface texture, homogeneity, and microstructural features among the unaged and aged binders. The resulting images were used to support the interpretation of the conventional and rheological responses and to provide morphological evidence of the influence of PPA concentration on the ageing behaviour of bitumen.

### 2.8. Fourier Transform Infrared Spectroscopy (FTIR)

The chemical characteristics of the control and PPA-modified bitumens were investigated using Fourier transform infrared (FTIR) spectroscopy was performed using a Nicolet 6700 FTIR spectrometer (Thermo Fisher Scientific, Madison, WI, USA)., to identify changes in the functional groups associated with PPA modification and short-term ageing. FTIR analysis was performed on both unaged and RTFO-aged binders to evaluate changes in the chemical characteristics of the bitumen before and after ageing. The FTIR spectra were recorded over a wavenumber range of 4000 to 400 cm^−1^. The spectra were evaluated based on the position and intensity of characteristic absorption bands to identify changes associated with increasing PPA concentration.

## 3. Result and Discussions

### 3.1. Conventional Properties of Modified Bitumen

#### 3.1.1. Penetration and Softening Point

**Figure 3:**
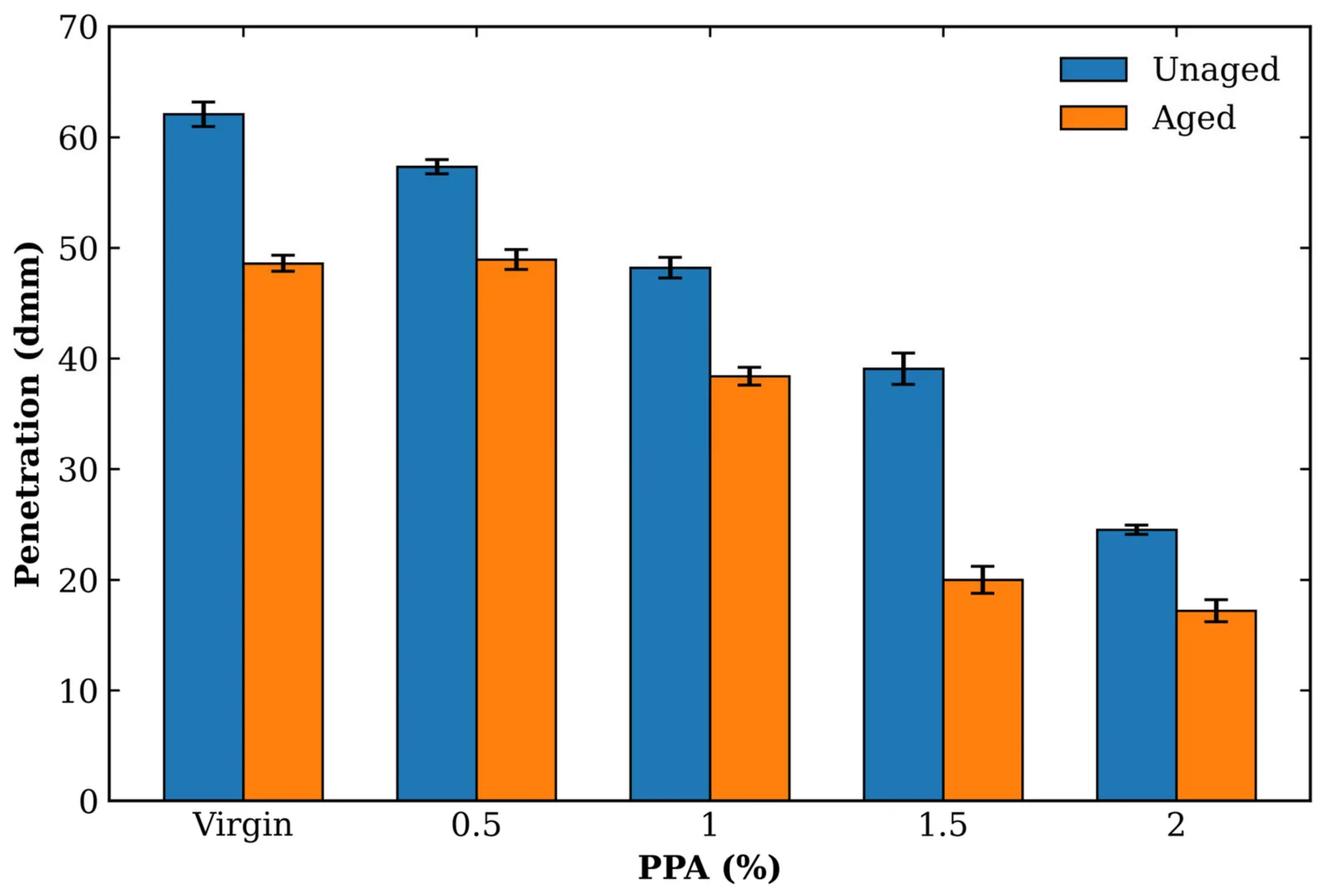
Penetration resistance of unaged and aged PPA-modified bitumens.

The penetration and softening point results demonstrate a clear influence of PPA concentration on the consistency and thermal response of the bitumen. As shown in Figure 3, the penetration of the unaged virgin bitumen was approximately 62 dmm, which progressively decreased to approximately 57, 48, 39, and 24 dmm with the addition of 0.5%, 1.0%, 1.5%, and 2.0% PPA, respectively. At 1.0% PPA, penetration decreased by approximately 23% relative to the virgin binder. The reduction became considerably more pronounced at higher PPA concentrations, indicating progressive hardening of the binder with increasing PPA content. Similar reductions in penetration with increasing PPA concentration have been reported in previous investigations [15,18]. In particular, Jiang et al. [11] observed a progressive reduction in penetration over a comparable PPA concentration range, confirming the strong influence of PPA dosage on binder consistency. The reduction in penetration can be associated with physicochemical interactions between PPA and the polar constituents of bitumen. Previous studies have reported that PPA can influence the association and distribution of asphaltenes and other polar fractions, which may alter the colloidal organisation of the binder [6,17]. Liu et al. [17] reported that PPA modification altered the colloidal structure of bitumen towards a more structured state, accompanied by changes in its rheological behaviour. Such structural rearrangement can restrict molecular mobility and consequently increase binder consistency. Masson (2008) [6] further demonstrated that the interaction between PPA and bitumen is strongly influenced by the chemical nature of the base bitumen, particularly its polar and reactive constituents. Therefore, the progressive reduction in penetration observed in the present study can reasonably be associated with PPA-induced physicochemical and structural changes within the binder rather than simply a physical thickening effect.

**Figure 4:**
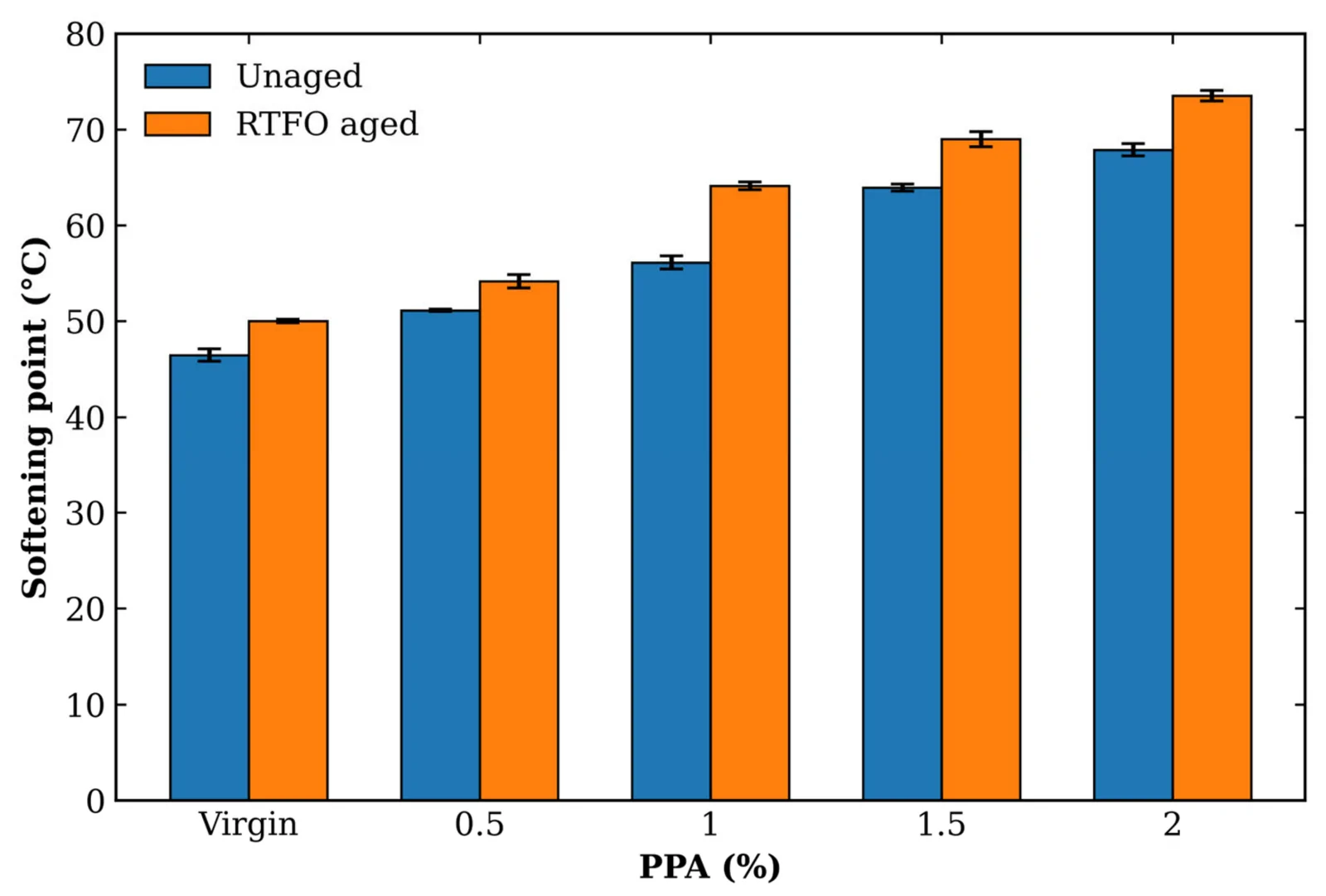
Softening point of unaged and aged PPA-modified bitumens.

The softening point exhibited an opposite trend as presented in Figure 4. The softening point of the unaged virgin bitumen was approximately 46.5 °C, which increased progressively to approximately 51, 56, 64, and 68 °C at PPA concentrations of 0.5%, 1.0%, 1.5%, and 2.0%, respectively. At 1.0% PPA, the softening point was approximately 20% higher than that of the virgin binder. A particularly pronounced increase occurred between 1.0% and 1.5% PPA, where the softening point increased from approximately 56 to 64 °C. Similar increases in softening point with PPA concentration have been reported in previous studies including that of Jiang et al. [11], who observed an increasing softening point with progressive PPA modification. From a mechanistic perspective, the increase in softening point is consistent with the structural changes responsible for the reduction in penetration. Interactions of PPA with the polar constituents of bitumen can modify the association of asphaltenes and the colloidal organisation of the binder, resulting in a more structured system with restricted molecular mobility [11,17]. Consequently, greater thermal energy is required to achieve sufficient molecular movement for the binder to soften and flow. The simultaneous reduction in penetration and increase in softening point therefore provide complementary macroscopic evidence of the stiffening effect associated with PPA modification. This behaviour is also consistent with previous studies in which PPA modification increased binder stiffness and improved resistance to flow at higher temperatures [19].

RTFO ageing caused further hardening of both the virgin and PPA-modified binders. Following ageing, the penetration of the virgin binder decreased from approximately 62 to 49 dmm. For the PPA-modified binders, penetration changed from approximately 57 to 49 dmm at 0.5% PPA, 48 to 38 dmm at 1.0% PPA, 39 to 20 dmm at 1.5% PPA, and 24 to 17 dmm at 2.0% PPA. The effect was particularly pronounced for the binder containing 1.5% PPA, indicating that the influence of PPA concentration on the penetration response following ageing was not proportional. The softening point results after RTFO ageing showed a corresponding increase. The softening point of the virgin binder increased from approximately 46.5 to 50 °C, while the aged binders containing 0.5%, 1.0%, 1.5%, and 2.0% PPA exhibited values of approximately 54, 64, 69, and 73.5 °C, respectively. Similar increases in binder consistency following RTFO ageing have been reported for PPA-modified bitumens [20]. Previous studies have also demonstrated that the influence of PPA on ageing behaviour depends on both PPA concentration and the chemical characteristics of the base bitumen [19]. The additional hardening following RTFO exposure can be associated with the physicochemical changes occurring during short-term ageing. Oxidation and the loss of lighter volatile constituents increase molecular association within bitumen, resulting in reduced penetration and increased softening point [13]. In PPA-modified bitumen, these ageing processes occur in a binder whose internal organisation has already been altered by PPA modification. Consequently, the combined response depends on both the initial structural effect of PPA, and the subsequent changes caused by ageing. Previous investigations of PPA-modified bitumen have similarly reported that PPA can alter the ageing response of the binder, although the magnitude of this effect varies with PPA concentration and base bitumen composition [21]. The present results support this observation, particularly because the penetration changes after ageing did not follow a simple proportional relationship with increasing PPA concentration. Overall, the penetration and softening point results consistently demonstrate that PPA progressively increased the consistency and resistance to thermal softening of the bitumen.

#### 3.1.2. Rotational Viscosity

**Figure 5:**
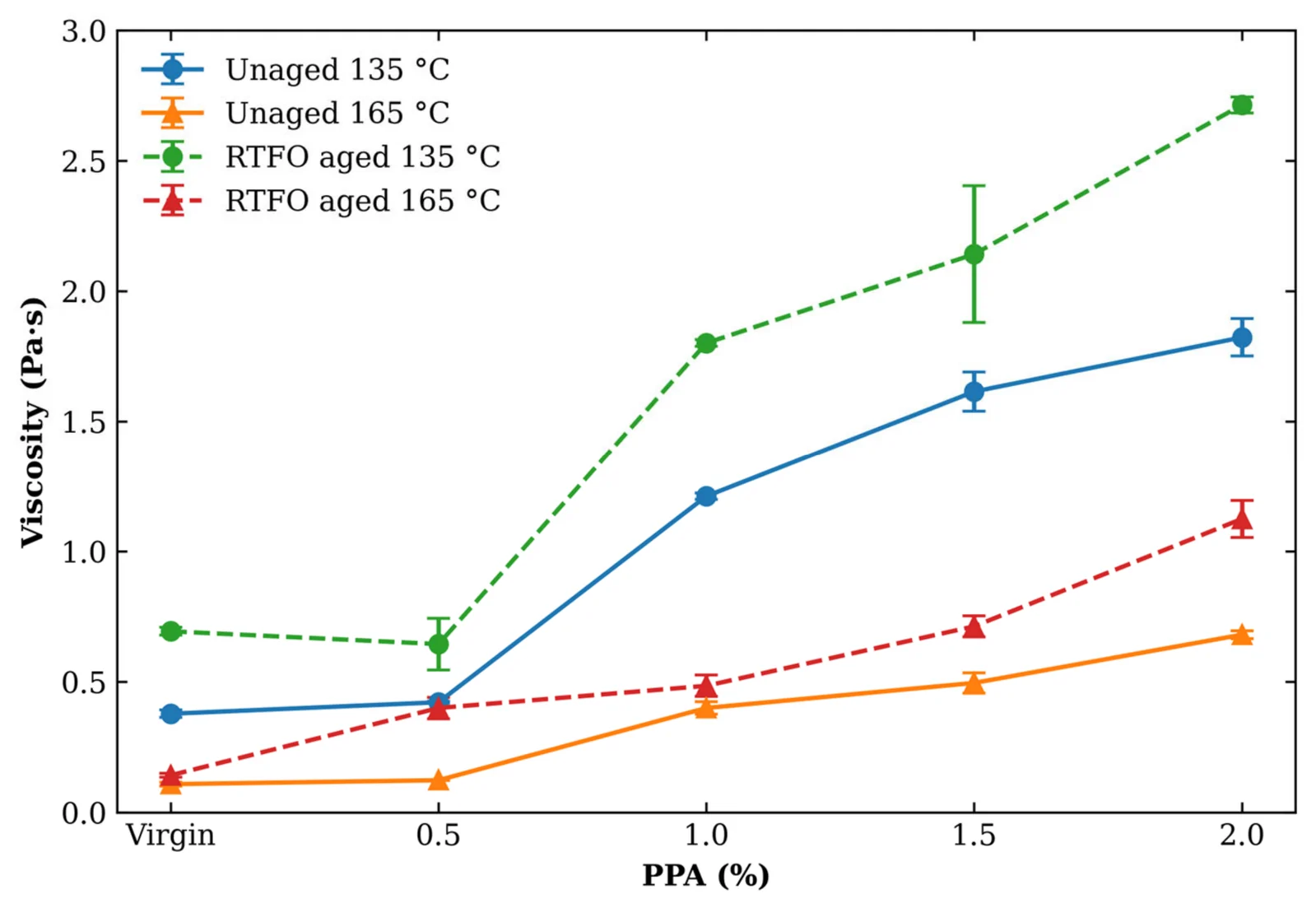
Rotational viscosity of unaged and aged PPA-modified bitumen at 135 °C and 165 °C.

The rotational viscosity results presented in Figure 5 reveal a concentration-dependent response to PPA modification, with a noticeable change in behaviour beyond the lowest PPA concentration. At 135 °C, the unaged virgin bitumen exhibited a viscosity of approximately 0.38 Pa·s, while the addition of 0.5% PPA produced only a small increase to approximately 0.42 Pa·s. A considerably stronger response occurred at 1.0% PPA, where the viscosity reached approximately 1.20 Pa·s, corresponding to an increase of approximately 216% relative to the virgin binder. Further increases to approximately 1.61 and 1.83 Pa·s were observed at 1.5% and 2.0% PPA, respectively. The relatively small change at 0.5%, followed by the pronounced increase at 1.0% and above, indicates that the effect of PPA on flow behaviour was not proportional throughout the investigated concentration range. Comparable increases in rotational viscosity with PPA addition have been reported in previous studies, including investigations conducted over similar PPA concentration ranges [11,18]. A dedicated investigation of the high temperature flow behaviour of PPA-modified bitumen also demonstrated that viscosity is strongly influenced by PPA concentration and testing temperature [15]. The increase in viscosity is consistent with physicochemical changes occurring within the bitumen following PPA modification. PPA can interact with polar constituents of bitumen and alter the association and distribution of asphaltenes, thereby modifying the colloidal organisation of the binder [6,17]. The resulting increase in intermolecular association and restriction of molecular mobility can increase resistance to shear flow, which is manifested macroscopically as higher rotational viscosity. Therefore, the viscosity increase observed in the present study is consistent with the reduction in penetration and increase in softening point discussed previously. However, rotational viscosity additionally demonstrates how these structural changes influence the flow characteristics of the binder at higher processing temperatures. The influence of temperature is clearly evident from the measurements at 165 °C, where substantially lower viscosity values were obtained for all modified bitumens. The viscosity of the unaged virgin binder was approximately 0.10 Pa·s, compared with approximately 0.12, 0.39, 0.48, and 0.67 Pa·s for 0.5%, 1.0%, 1.5%, and 2.0% PPA, respectively. Increasing temperature provides greater thermal energy for molecular movement and consequently reduces resistance to flow. Nevertheless, the separation between the virgin binder and binders containing 1.0% PPA or more remained evident at 165 °C. This indicates that the influence of PPA on binder structure persisted even at a temperature where the binder was substantially more fluid. Similar temperature-dependent viscosity behaviour has been reported for PPA-modified bitumens, where increasing temperature reduced viscosity while the relative influence of PPA concentration remained apparent [20].

RTFO ageing produced a further change in flow behaviour. At 135 °C, the aged virgin binder exhibited a viscosity of approximately 0.69 Pa·s, while values of approximately 0.64, 1.82, 2.14, and 2.71 Pa·s were obtained for binders containing 0.5%, 1.0%, 1.5%, and 2.0% PPA, respectively. The additional resistance to flow following RTFO exposure is consistent with the hardening associated with short-term ageing. Oxidative processes and changes in the lighter fractions of bitumen during ageing promote greater molecular association and reduce molecular mobility, thereby increasing viscosity. Previous investigations have similarly reported increased viscosity and rheological stiffness of PPA-modified binders following RTFO ageing, although the extent of this change depends on PPA concentration and the characteristics of the base bitumen [4]. An interesting response was observed at 0.5% PPA, where the aged viscosity at 135 °C was approximately 0.64 Pa·s, slightly lower than the 0.69 Pa·s of the aged virgin binder. Together with the small difference between the corresponding unaged binders, this suggests that 0.5% PPA produced only a limited change in flow behaviour. In contrast, the pronounced increase from 1.0% PPA onwards indicates a stronger modification response. Since the effectiveness of PPA depends on both dosage and the chemical characteristics of the base bitumen [6], this apparent transition should be further examined through the rheological and physicochemical results rather than interpreted as a definite modification threshold. At 165 °C, the aged viscosities were approximately 0.14, 0.39, 0.48, 0.70, and 1.12 Pa·s for the virgin, 0.5%, 1.0%, 1.5%, and 2.0% PPA binders, respectively. Although the higher temperature substantially reduced resistance to flow, the effect of ageing varied among the PPA concentrations. This confirms that the ageing response was dependent on both PPA concentration and testing temperature rather than following a uniform trend, consistent with previous observations for PPA-modified bitumen [4]. Despite the increase in viscosity, all values measured at 135 °C remained below 3 Pa·s, with the maximum value of approximately 2.71 Pa·s recorded for the aged 2.0% PPA binder. Thus, even the aged specimens remained below the commonly referenced Superpave viscosity criterion of 3 Pa·s at 135 °C for the unaged binder. Nevertheless, the increasing viscosity at higher PPA concentrations indicates greater resistance to flow and should be considered when assessing the overall effect of PPA modification.

Overall, the viscosity results reveal a limited response at 0.5% PPA followed by a considerably stronger effect from 1.0% PPA onwards. This behaviour remained evident at both temperatures, while RTFO ageing further demonstrated that the response depended on PPA concentration. The observed changes are consistent with reported interactions of PPA with the polar constituents and asphaltene structure of bitumen [6,17]. The subsequent rheological analysis provides a more direct basis for determining whether these changes in flow behaviour correspond to broader changes in the viscoelastic characteristics of the binder. The ageing results presented in this study represent the short-term response of the binders under RTFO conditions. Long-term ageing may result in different rheological and physicochemical changes and requires further investigation under extended ageing conditions. Statistical analysis further confirmed that PPA dosage significantly affected all the evaluated conventional properties (*p* < 0.001). The mean values, standard deviations, and Tukey’s HSD comparisons are presented in Table 2. Significant differences were observed among most PPA dosages, while some lower dosage groups showed no significant differences, particularly for rotational viscosity. The ageing results presented in this study represent the short-term response of the binders under RTFO conditions. Long-term ageing may result in different rheological and physicochemical changes and requires further investigation under extended ageing conditions.

#### 3.1.3. Temperature Susceptibility and Ageing Indices

The temperature susceptibility of the binders was evaluated using the penetration index, PI, and penetration viscosity number, PVN, as presented in Table 3. The ideal PI range is reported between −1 and +1 for better performing pavements [22]. The virgin bitumen exhibited a PI of −1.64, which lies outside this range and indicates relatively high temperature susceptibility. With PPA modification, PI increased to −0.61, 0.13, 1.18, and 0.86 at 0.5%, 1.0%, 1.5%, and 2.0% PPA, respectively. Thus, 0.5%, 1.0%, and 2.0% PPA brought the PI within the ideal range, whereas the value of 1.18 at 1.5% PPA was slightly above its upper limit. The substantial shift from −1.64 for the virgin binder to values closer to zero and within the ideal range demonstrates that PPA effectively reduced the temperature susceptibility of the bitumen. A similar improvement in PI following PPA addition was also reported by [21]. PVN provided a similar indication of the change in temperature susceptibility. The PVN generally ranges from +0.5 to −2.0, with larger positive values representing lower temperature susceptibility. The PVN increased from −0.79 for the virgin binder to −0.71, 0.46, 0.58, and 0.28 with increasing PPA concentration. The considerable shift towards positive values, particularly at 1.0% and 1.5% PPA, confirms a substantial reduction in temperature susceptibility. The value of 0.58 at 1.5% PPA slightly exceeded the upper reference value of +0.5, while the value decreased to 0.28 at 2.0% PPA. The PI and PVN results therefore indicate that increasing PPA did not produce a continuously proportional improvement, despite considerably improving the temperature susceptibility compared with the virgin binder.

The improvement can be associated with the interaction of PPA with the bitumen constituents. Previous studies have associated the reduction in temperature susceptibility with interactions between PPA and bitumen constituents, including changes involving the asphaltene and resin fractions and the colloidal organisation of the binder [23]. Such changes may restrict molecular mobility and reduce the sensitivity of the binder to temperature. Hazoor Ansari et al. [21] similarly attributed the improvement produced by PPA to its interaction with bitumen and the resulting structural changes. This interpretation is also supported by the mechanisms reported by Liu et al. [17].

The ageing indices present a more complex response. PAI values were 0.78, 0.86, 0.79, 0.51, and 0.69 for the virgin, 0.5%, 1.0%, 1.5%, and 2.0% PPA binders, respectively. As PAI represents the retention of penetration after RTFO ageing, a value closer to unity corresponds to a smaller relative change in penetration. The highest PAI of 0.86 at 0.5% PPA therefore indicates the greatest penetration retention, whereas the markedly lower value of 0.51 at 1.5% PPA reflects considerably greater hardening. The recovery to 0.69 at 2.0% PPA further demonstrates that the penetration-based ageing response did not vary systematically with increasing PPA concentration. In comparison, SAI was considerably less sensitive to PPA concentration. The values remained between 1.06 and 1.14, with values of 1.08, 1.06, 1.14, 1.08, and 1.08 obtained for the respective binders. This narrow variation indicates that the relative increase in softening point caused by RTFO ageing remained broadly similar despite the substantial differences observed in penetration. Consequently, PAI and SAI provide somewhat different perspectives on ageing, highlighting the limitation of assessing ageing susceptibility from a single conventional property.

A clearer influence of PPA was evident in VAI at 135 °C. The VAI decreased from 1.84 for the virgin binder to 1.53, 1.49, 1.33, and 1.49 at 0.5%, 1.0%, 1.5%, and 2.0% PPA, respectively. Since values closer to unity represent a smaller proportional increase in viscosity following ageing, these results suggest that PPA generally reduced the viscosity-based ageing susceptibility at 135 °C. The lowest VAI of 1.33 occurred at 1.5% PPA. Interestingly, this concentration simultaneously produced the lowest PAI. Therefore, 1.5% PPA appeared relatively favourable when ageing was evaluated through viscosity but comparatively susceptible when evaluated through penetration. This contrasting behaviour demonstrates that the influence of PPA on ageing cannot be adequately represented by a single conventional ageing index. The response at 165 °C was less systematic, with VAI values of 1.31, 3.26, 1.21, 1.46, and 1.64, respectively. The exceptionally high value of 3.26 at 0.5% PPA reflects the large relative change from its low initial viscosity following RTFO ageing, whereas the lowest value of 1.21 was obtained at 1.0% PPA. The strong variation with testing temperature indicates that viscosity-based ageing susceptibility is affected by both PPA concentration and the temperature at which the flow response is evaluated. Previous investigations have similarly demonstrated that the short-term ageing behaviour of PPA-modified bitumen depends on PPA dosage, binder characteristics, and the property selected for ageing assessment [15,24].

The concentrations of the PPA in the bitumen indicate the differences in the ageing indices which ultimately reflect the response of the modified bitumens. Recent studies have shown that rejuvenation reinstates the viscoelastic characteristics of bitumen by variations in the molecular interactions of components of bitumen [25]. These outcomes are very relevant to the bitumen modified with PPA as the variation in the indices’ responses seen at various PPA dosages may also affect its successive response to any rejuvenation. Therefore, further studies are recommended to assess how adding PPA mainly affects the restoration of the modified aged bitumen.

### 3.2. Dynamic Rheological Properties

Bitumen exhibits viscoelastic behaviour that is strongly influenced by temperature and loading frequency. In this study, the rheological behaviour of unaged and RTFO-aged PPA-modified bitumens was evaluated using the rutting parameter, frequency sweep, master curves, Black space diagram and Cole–Cole plots to assess high temperature performance and overall viscoelastic response.

#### 3.2.1. Rutting Parameter and Performance Grade

**Figure 6:**
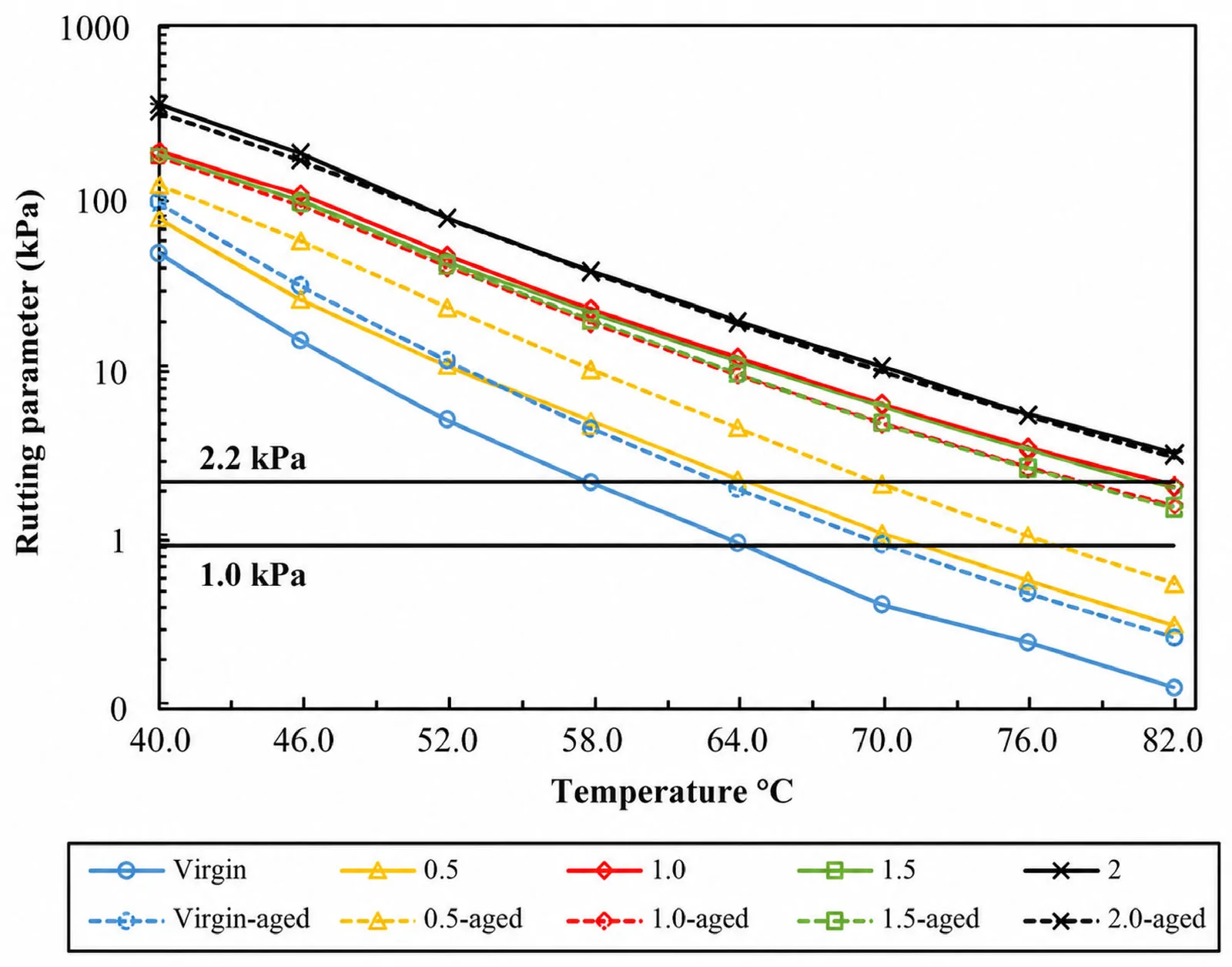
Rutting parameter of unaged and aged modified bitumens.

The rutting parameter, G*/sinδ, was used to evaluate the high temperature deformation resistance of the virgin and PPA-modified bitumens. Higher G*/sinδ values indicate greater resistance to permanent deformation. As shown in Figure 6, G*/sinδ decreased continuously with increasing temperature for all binders. However, the incorporation of PPA shifted the curves towards higher values, with a particularly pronounced improvement at PPA concentrations of 1.0% and above. Similar improvements in the high temperature rheological response of PPA-modified bitumen have been reported in previous studies [11,12]. The rheological parameters and corresponding high temperature performance grades are summarised in Table 4. For the unaged binders, G*/sinδ increased from 1.01 kPa for the virgin bitumen to 2.37, 12.35, 11.93, and 20.73 kPa with 0.5%, 1.0%, 1.5%, and 2.0% PPA, respectively. The relatively small change at 0.5% followed by the substantial increase at 1.0% indicates a nonlinear response to PPA concentration. The highest value was obtained at 2.0% PPA. This behaviour has been associated with changes in the colloidal organisation and rheological structure of bitumen following PPA modification [17,26].

The phase angle also decreased substantially with increasing PPA concentration. As presented in Table 4, δ decreased from 88.70° for the virgin binder to 81.00° at 0.5% PPA and approximately 61° at 1.0% and 1.5% PPA, reaching 55.10° at 2.0% PPA. The reduction in δ indicates an increasing elastic contribution to the binder response. Therefore, the improvement in G*/sinδ resulted from both increased stiffness and a change in the viscoelastic behaviour of the bitumen. Similar reductions in phase angle with PPA modification have been observed in previous rheological investigations [12,17]. The improvement in high temperature performance was further reflected by the failure temperature. The virgin binder exhibited a failure temperature of 64.2 °C, while 0.5% PPA increased it to 72.4 °C. For PPA concentrations of 1.0% and above, the failure temperature exceeded the maximum investigated temperature of 82 °C. Consequently, the high temperature grade increased from PG 64 for the virgin binder to PG 70 at 0.5% PPA and PG 82 at PPA concentrations of 1.0% and above. The results therefore indicate that the most pronounced improvement in high temperature performance occurred between 0.5% and 1.0% PPA. Following RTFO ageing, the PPA-modified binders continued to exhibit substantially higher G*/sinδ and lower δ than the virgin binder. The G*/sinδ values were 2.08, 4.79, 9.92, 9.92, and 19.36 kPa for the virgin, 0.5%, 1.0%, 1.5%, and 2.0% binders, respectively. The corresponding phase angles decreased from 87.30° for the aged virgin binder to 53.20° at 2.0% PPA. The failure temperatures of the aged virgin and 0.5% PPA binders were approximately 64 and 69 °C, respectively, whereas the binders containing 1.0% PPA and above maintained failure temperatures greater than 82 °C. These results demonstrate that the beneficial influence of PPA on high temperature rheological behaviour was retained following short-term ageing. Previous studies have also reported that PPA significantly affects the rheological response of bitumen following RTFO ageing [4].

Overall, the results from Figure 6 and Table 4 demonstrate that PPA substantially improved the high temperature deformation resistance of the bitumen. A marked transition was observed at approximately 1.0% PPA, beyond which the binders maintained failure temperatures above 82 °C. Although 2.0% PPA produced the highest G*/sinδ and lowest phase angle, the high temperature grade did not increase beyond that obtained at 1.0% PPA. This suggests that the additional stiffening obtained at higher PPA concentrations did not provide a corresponding improvement in the measured performance grade.

#### 3.2.2. Master Curves

**Figure 7:**
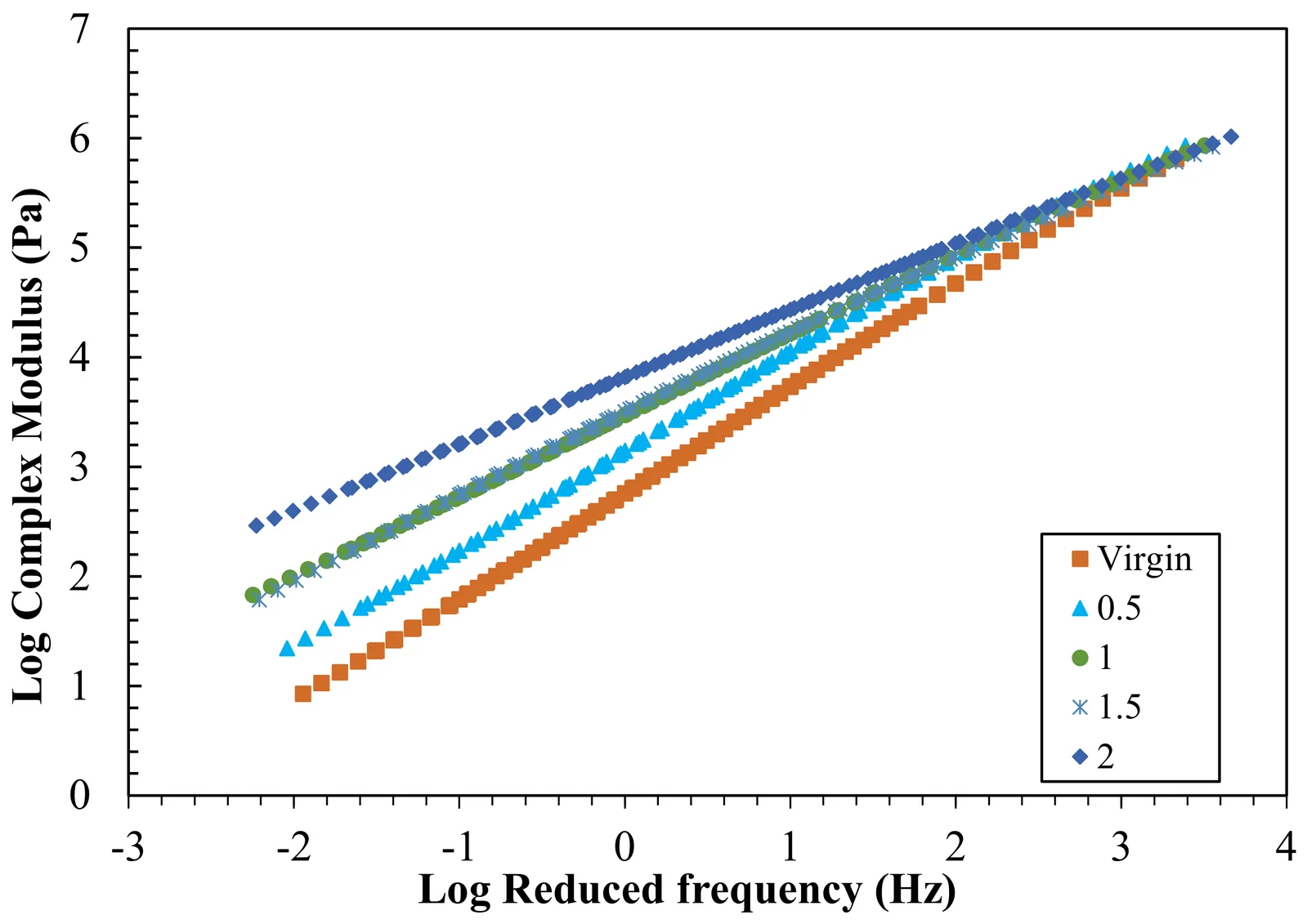
Master curve of unaged PPA-modified bitumens.

**Figure 8:**
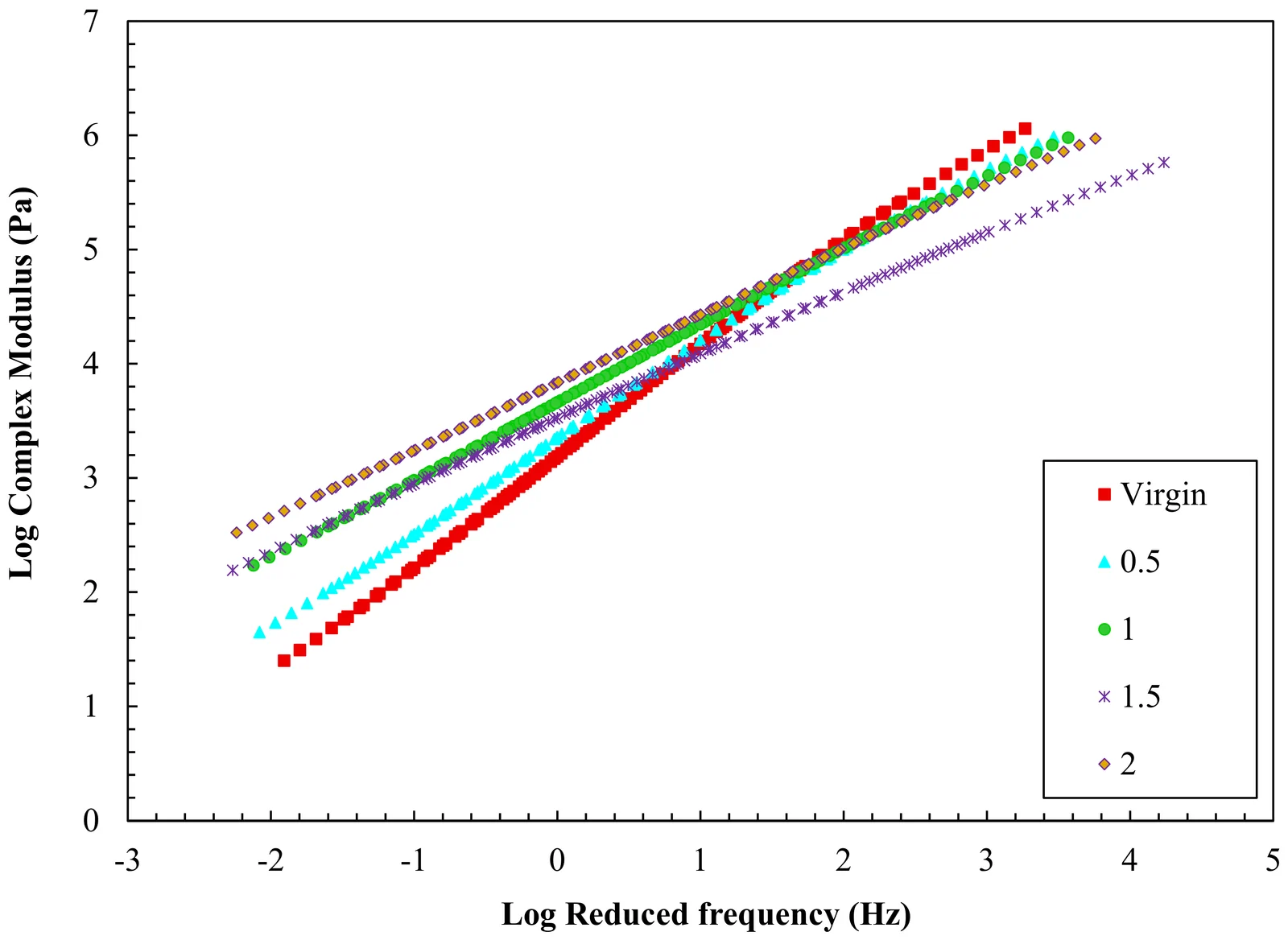
Master curve of aged PPA-modified bitumens.

The master curves of the virgin bitumen and binders containing 0.5%, 1.0%, 1.5%, and 2.0% PPA, before and after RTFO ageing, are presented in Figure 7 and Figure 8, respectively. The curves were constructed at a reference temperature of 65 °C using the time temperature superposition principle. Accordingly, the lower reduced frequency region represents higher temperature and longer loading conditions, whereas the higher reduced frequency region corresponds to lower temperature and shorter loading conditions. Master curves extend the experimentally accessible frequency range and provide a useful representation of the frequency-dependent viscoelastic response of modified bitumen [15]. For the unaged binders, the curves show a clear distinction among the different PPA concentrations, particularly at lower reduced frequencies. The virgin binder exhibited the lowest G*, followed by 0.5% PPA, while a substantial upward shift occurred at 1.0% PPA. The responses of 1.0% and 1.5% PPA were relatively close, whereas 2.0% PPA maintained the highest G* in this region. This pattern indicates a nonlinear concentration effect, where the rheological change between 0.5% and 1.0% PPA was more pronounced than that between 1.0% and 1.5%. A stronger influence of PPA in the low frequency region has also been observed in frequency sweep studies, where increased PPA concentration produced greater changes in complex modulus under conditions corresponding to higher temperature and longer loading time [27].

With increasing reduced frequency, however, the differences among the unaged binders gradually diminished and the curves approached one another. The modification effect was therefore not uniform throughout the frequency spectrum. This convergence suggests that the influence of PPA on G* becomes less dominant towards conditions representing lower temperatures and shorter loading times. Similar master curve behaviour has been reported for PPA-containing binders, where the increase in G* was more pronounced at lower frequencies and comparatively smaller at higher frequencies [13].

The master curves after RTFO ageing, presented in Figure 8, show a distinctly different pattern. At lower reduced frequencies, the PPA-modified binders remained above the virgin binder, with greater separation becoming evident at concentrations of 1.0% and above. As the reduced frequency increased, however, the curves converged and subsequently changed their relative positions. At the upper frequency region, the virgin binder exhibited comparatively higher G*, while the 1.5% PPA binder showed the lowest response among the investigated binders. The occurrence of curve convergence and crossing indicates that RTFO ageing altered the frequency dependence of the binders rather than producing an equivalent shift over the entire master curve. This frequency-dependent ageing response provides additional information to the conventional ageing indices discussed in Section 3.1.3. Those indices showed that no single PPA concentration consistently provided the lowest ageing susceptibility across all conventional properties. The master curves extend this observation by demonstrating that the relative effect of ageing also changes according to loading frequency. Such behaviour is reasonable for PPA-modified bitumen because its rheological response is influenced by PPA concentration, temperature, and loading conditions, while the magnitude of these effects can vary across the rheological domain [28].

Overall, the master curves reveal that the influence of PPA is most distinguishable in the lower reduced frequency region, while the differences diminish or change towards higher frequencies, particularly after RTFO ageing. The 0.5% PPA produced a moderate shift, the most noticeable change occurred at 1.0% PPA, the response at 1.5% PPA remained relatively close to 1.0% over much of the unaged frequency range, and 2.0% PPA produced the greatest modulus at lower frequencies. More importantly, the altered curve ordering after ageing demonstrates that PPA concentration affects the frequency dependence of the rheological response, rather than simply increasing G* by a constant amount. This aspect is examined further using the Black and Cole–Cole representations.

#### 3.2.3. Black Space Diagram

**Figure 9:**
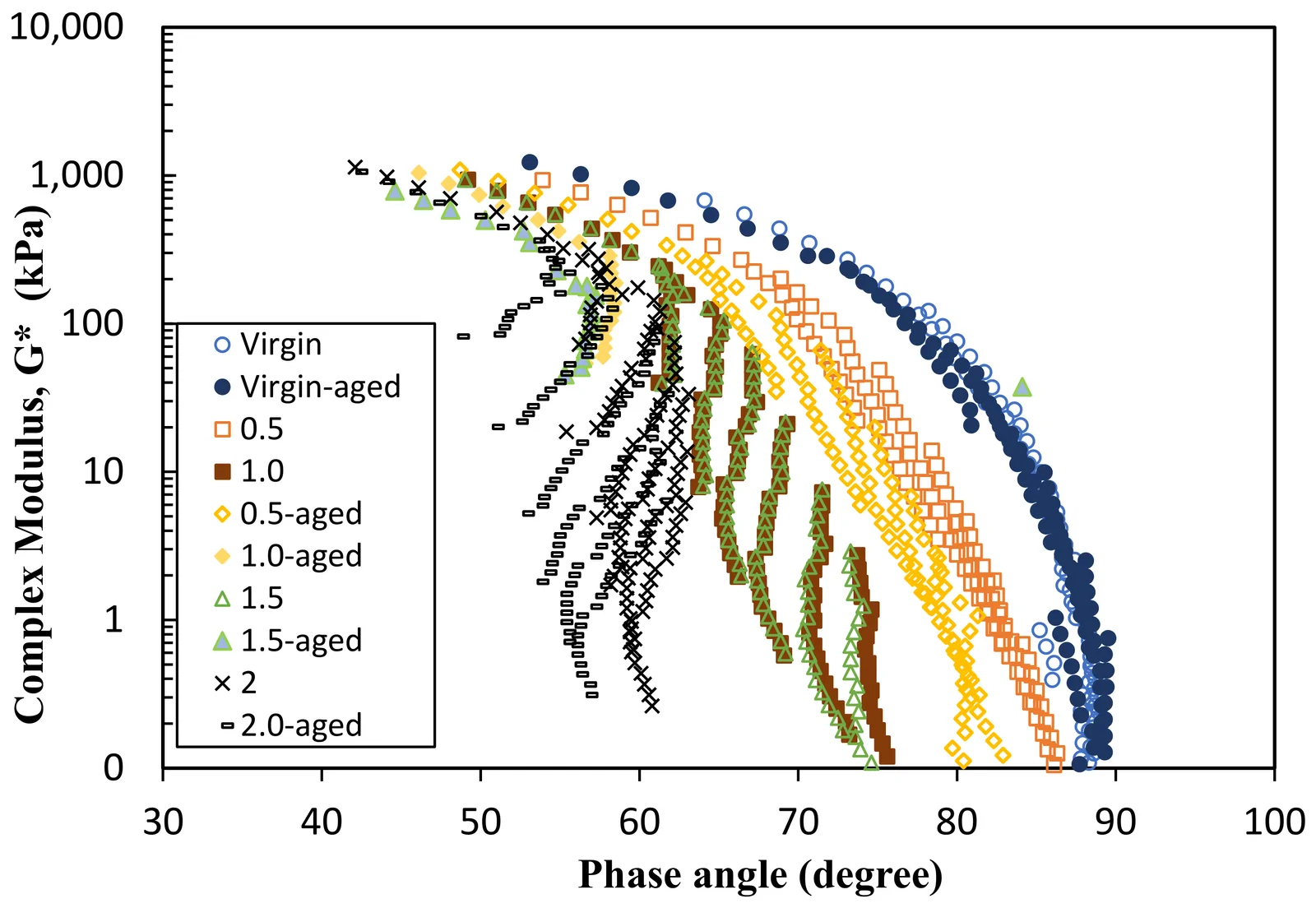
Black space diagram for unaged and aged PPA-modified bitumens.

The Black diagram, representing the relationship between complex modulus, G*, and phase angle, δ, for the virgin and PPA-modified bitumens in both unaged and RTFO-aged conditions, is presented in Figure 9. This representation allows the rheological response to be examined by directly relating binder stiffness to its viscous and elastic character. In general, a shift towards lower δ at a given G* indicates a greater elastic contribution to the binder response. A clear displacement of the PPA-modified binders towards lower phase angles can be observed compared with the virgin bitumen. The virgin binder is predominantly located in the higher δ region and approaches 90° as G* decreases, reflecting an increasingly viscous response under softer testing conditions. Addition of 0.5% PPA produces a noticeable displacement towards lower δ, while a substantially greater shift occurs when the PPA concentration reaches 1.0%. The responses at 1.0% and 1.5% PPA occupy broadly similar regions of the Black space, whereas 2.0% PPA extends further towards lower phase angles. The progressive displacement of the PPA-modified binders towards lower phase angles indicates an increasing elastic contribution to the rheological response. Similar changes in Black space have been reported for PPA-modified binders, where increasing PPA concentration altered the relationship between G* and δ and produced a more pronounced rheological response than that of the virgin binder [29,30]. The substantial change observed around 1.0% PPA in the present study is also consistent with previous observations that the rheological influence of PPA may become pronounced within relatively low modification levels rather than increasing proportionally with concentration [29].

The separation among the PPA concentrations is particularly evident in the intermediate and lower phase angle regions. Compared with 0.5% PPA, the pronounced displacement observed at 1.0% indicates a substantial change in rheological character at this concentration. Increasing the concentration to 1.5% produces a comparatively smaller additional shift, whereas the 2.0% binder extends to the lowest δ values observed in the Black space. This behaviour agrees with the phase angle results reported in Section 3.2.1, where δ decreased considerably with progressive PPA addition. The Black diagram, however, demonstrates that this change occurs over a broad range of G* rather than at only a single temperature or loading condition. The combined representation further provides a direct comparison of the binders before and after RTFO ageing. Ageing changes both the position and trajectory of the Black curves, although the magnitude of this change varies with PPA concentration. The aged virgin binder remains predominantly within the higher phase angle region, while the PPA-modified binders generally retain their displacement towards lower δ. In particular, the binders containing 1.0%, 1.5%, and 2.0% PPA continue to occupy considerably lower phase angle regions after RTFO ageing. This suggests that the modification of viscoelastic character produced by PPA persists following short-term ageing. Another notable feature is the variation in the shape and relative position of the trajectories after RTFO ageing. The unaged and aged responses do not simply overlap through a uniform increase in G*. Instead, their relative positions vary across the Black space, indicating that ageing modifies the relationship between G* and δ itself. The effect is also concentration-dependent, which is consistent with the ageing indices presented in Section 3.3, where the influence of PPA on short-term ageing varied according to the property considered. Thus, the Black diagram provides additional rheological evidence that the interaction between PPA modification and ageing is more complex than a simple stiffening process. These changes also complement the master curve behaviour discussed in Section 3.2.2. The convergence and change in relative positions observed in the master curves after RTFO ageing indicated a frequency-dependent modification of binder response. The Black diagram extends this interpretation by demonstrating corresponding changes in the relationship between stiffness and phase angle. Therefore, the differences observed after ageing can be attributed not only to changes in the magnitude of G*, but also to changes in the relative viscous and elastic contributions to the overall rheological response. Overall, the Black diagram confirms a progressive alteration in the viscoelastic character of bitumen with PPA modification. The subsequent Cole–Cole analysis further examines this behaviour through the relationship between the elastic and viscous components of the complex modulus.

#### 3.2.4. Cole–Cole Diagram

**Figure 10:**
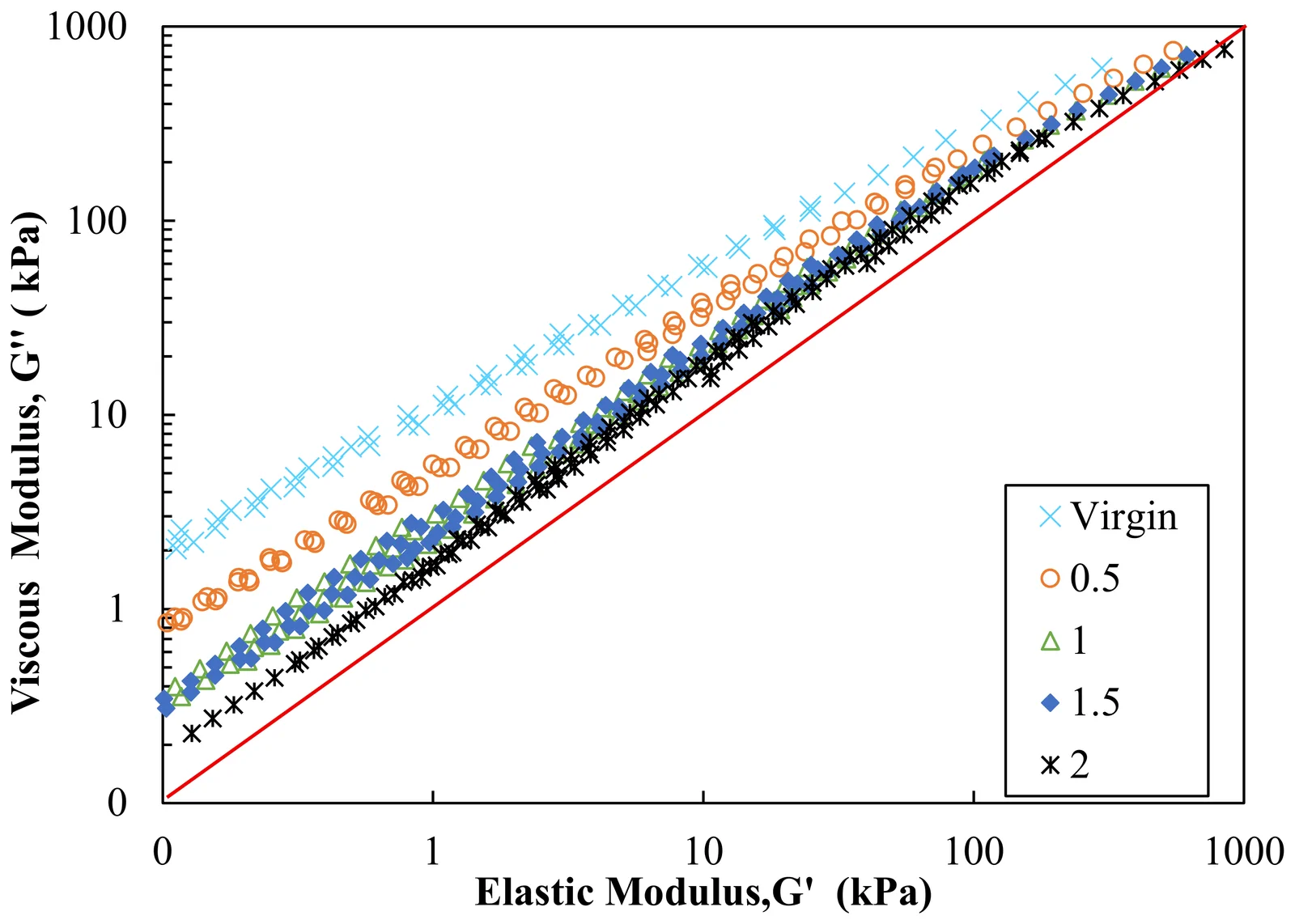
Cole–Cole diagram of unaged PPA-modified bitumens.

**Figure 11:**
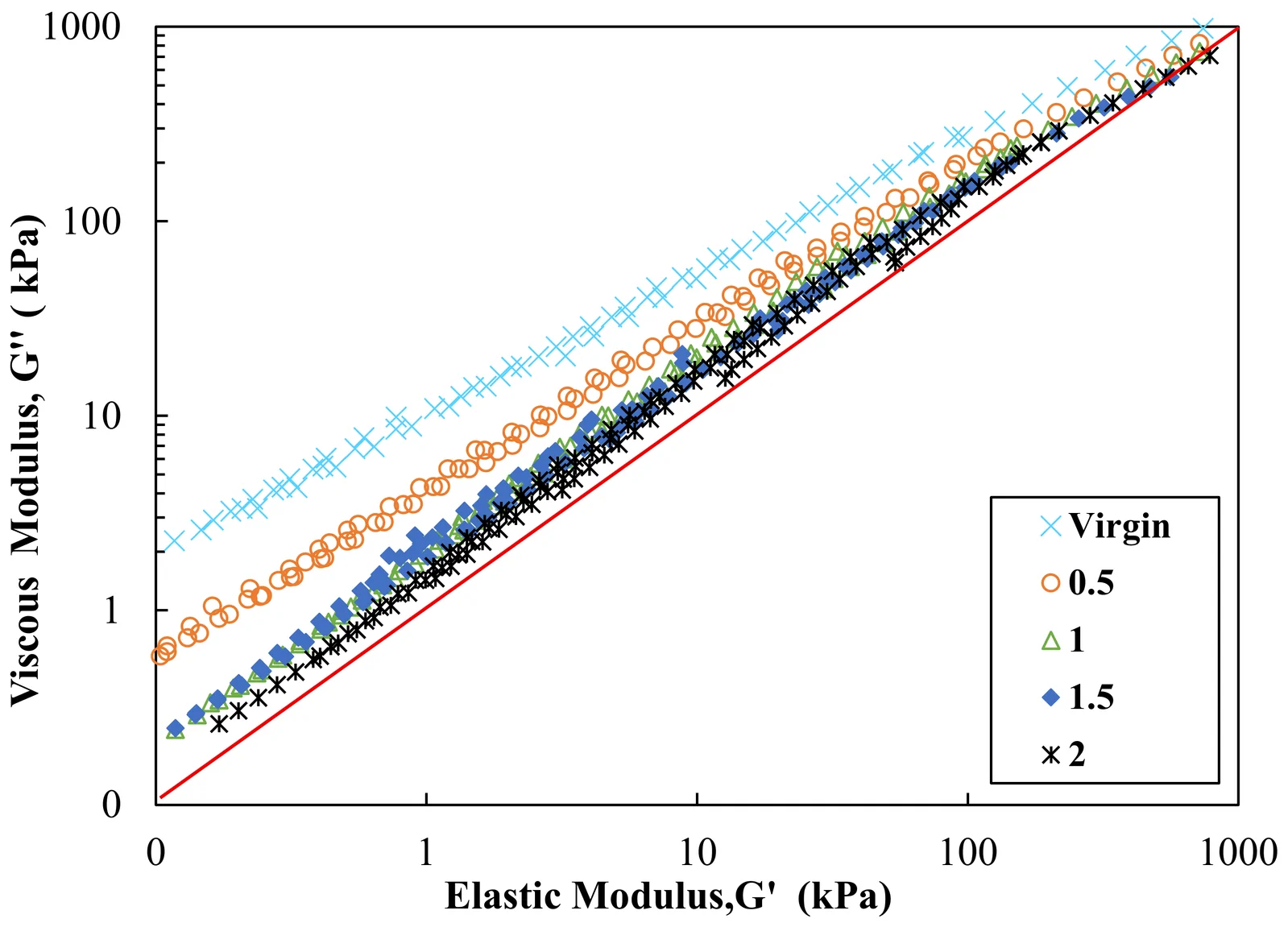
Cole–Cole diagram of aged PPA-modified bitumens.

The Cole–Cole diagrams of the unaged and aged PPA-modified bitumens are presented in Figure 10 and Figure 11, respectively. The relationship between elastic modulus, G′, and viscous modulus, G″, provides insight into the relative elastic and viscous contributions to the rheological response. Cole–Cole representation has previously been applied to PPA-modified binders to characterise changes in their viscoelastic behaviour [30,31]. For the unaged binders, the virgin bitumen exhibited the highest G″ at comparable G′ values, while PPA addition progressively shifted the response downwards. The change from 0.5% to 1.0% PPA was particularly evident, whereas the curves for 1.0%, 1.5%, and 2.0% were relatively closer. This indicates that PPA changes the balance between the viscous and elastic components, with a greater relative elastic contribution at higher PPA concentrations. Similar changes in the Cole–Cole response with PPA concentration have been attributed to alteration of the viscoelastic constituents of the modified binder [13].

Following RTFO ageing, the general distinction between the virgin and PPA-modified binders remained evident. The virgin binder continued to show comparatively higher G″ values, while the curves containing 1.0%, 1.5%, and 2.0% PPA remained relatively close over much of the investigated G′ range. This suggests that the altered viscoelastic balance produced by PPA persists after short-term ageing. The tendency of the curves to converge at higher G′ values further indicates that the magnitude of the PPA effect varies across the rheological domain rather than remaining constant. These results complement the Black diagram in Section 3.2.3, where PPA modification shifted the response towards lower phase angles. The Cole–Cole analysis provides further evidence that this behaviour results from a redistribution between the elastic and viscous components rather than simply an increase in overall stiffness. The relatively smaller differences among concentrations above 1.0% PPA also support the concentration-dependent transition observed in the preceding rheological analyses.

While considering the combined results of the master curve, the Black diagram and the Cole–Cole diagram, the observed curve intersections after the ageing of the bitumens can be further justified by the variations in the viscoelastic balancing of the modified binders across temperature and the frequency domains. The PPA-modified bitumens significantly retained a higher G* at reduced frequencies corresponding to higher temperatures and longer loading times. On the contrary, master curves changed their relative ordering and converged at higher reduced frequencies. After the short-term ageing G* and δ showing the Black space diagram were changed rather than demonstrating a uniform enhancement in the stiffness of the PPA-modified bitumens. These results are further supported by the Cole–Cole diagram where the variations in the relationship between elastic and viscous components in high temperature convergence demonstrate that the contribution of each component varies across the rheological domain. Consequently, after ageing the curve intersections reflect the temperature and frequency-dependent restructuring of the bitumen viscoelastic response and the stiffness of this change depends on the PPA concentration.

### 3.3. FESEM Analysis

**Figure 12:**
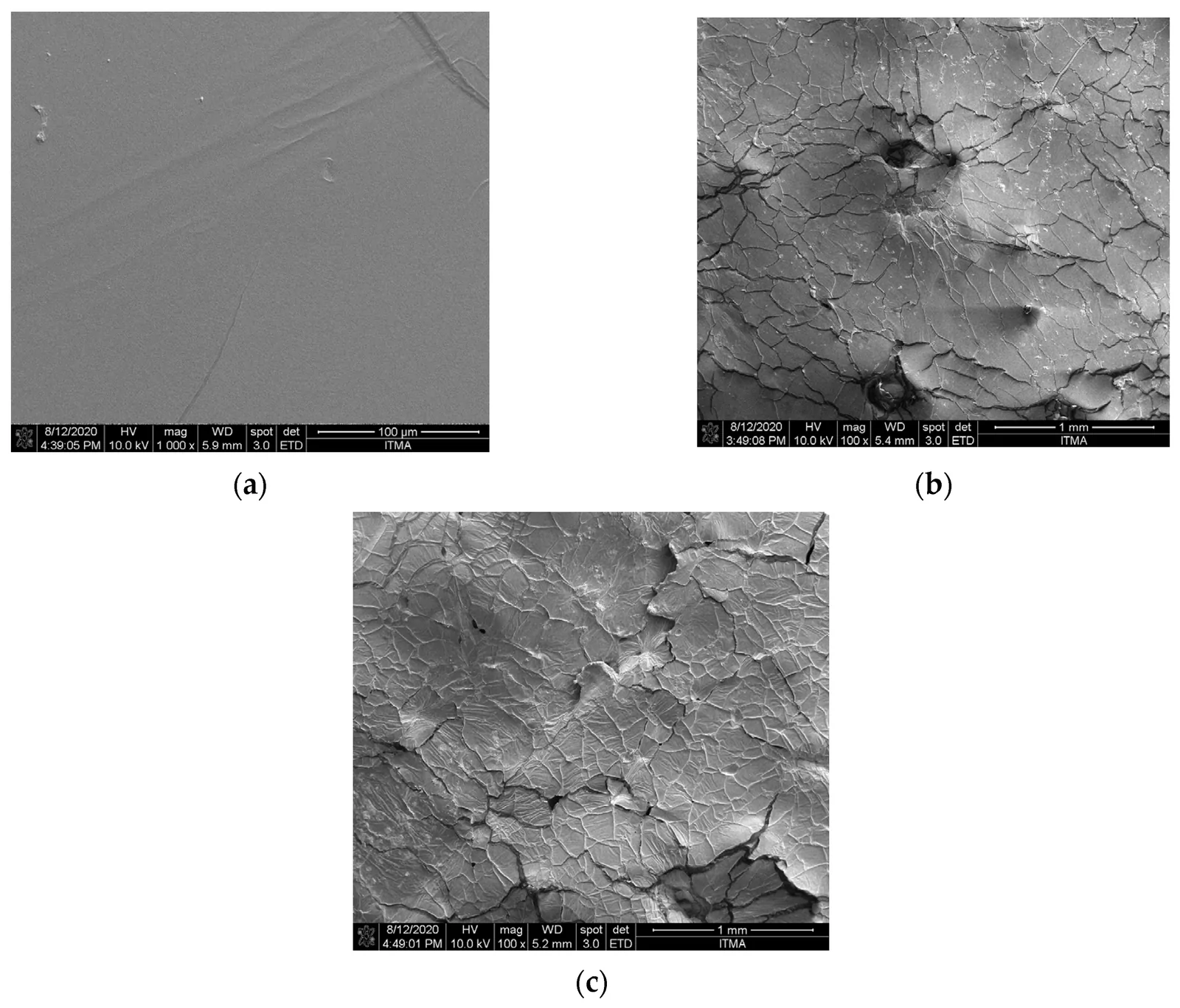
FESEM images of (**a**) virgin, (**b**) unaged, and (**c**) aged PPA-modified bitumen.

The surface morphology of the virgin, 1.0% PPA-modified, and corresponding RTFO-aged bitumens is presented in Figure 12. The virgin bitumen was included as a reference for identifying the morphological changes associated with PPA modification and subsequent ageing. Following the incorporation of 1.0% PPA, the binder retained a generally continuous and homogeneous appearance, without visible separation or distinct PPA-rich domains. This behaviour is reasonable considering the liquid nature and relatively low concentration of PPA, which allows it to be dispersed effectively throughout the bitumen during mixing. Previous microscopic investigations have similarly reported relatively uniform PPA-modified bitumen and demonstrated that the morphological response to PPA depends strongly on the composition and colloidal characteristics of the base bitumen [32,33].

Despite the absence of obvious phase separation, the 1.0% PPA-modified bitumen exhibits a more developed and textured surface morphology compared with the virgin binder. The appearance of finer irregular surface features suggests that modification has altered the internal organisation of the binder, although the FESEM image alone cannot establish the underlying chemical mechanism. Yan et al. reported that PPA-induced morphological changes were correlated with changes in the physical properties and chemical composition of bitumen, while more recent microscopic investigations have related PPA modification to changes in asphaltene dispersion and colloidal organisation [33,34]. These observations are consistent with the substantial changes in viscosity and rheological response observed in the present study at 1.0% PPA.

The morphological changes become more pronounced following RTFO ageing, with the aged PPA-modified binder showing a rougher, more irregular and more strongly developed surface texture than its unaged counterpart. This can reasonably be associated with the additional hardening and structural rearrangement occurring during short-term ageing. The observation is consistent with the rheological results, where the modified binder retained a distinctly altered viscoelastic response following RTFO ageing. Therefore, the FESEM results provide complementary morphological evidence that the increase in stiffness following PPA modification and subsequent ageing is accompanied by changes in the microscopic surface structure rather than visible segregation of the modifier. The morphological changes become more pronounced following RTFO ageing, with the aged PPA-modified binder showing a rougher, more irregular and more strongly developed surface texture than its unaged counterpart. This can reasonably be associated with the additional hardening and structural rearrangement occurring during short-term ageing. The observation is consistent with the rheological results, where the modified binder retained a distinctly altered viscoelastic response following RTFO ageing. Therefore, the FESEM results provide complementary morphological evidence that the increase in stiffness following PPA modification and subsequent ageing is accompanied by changes in the microscopic surface structure rather than visible segregation of the modifier.

### 3.4. FTIR of Modified Bitumens

**Figure 13:**
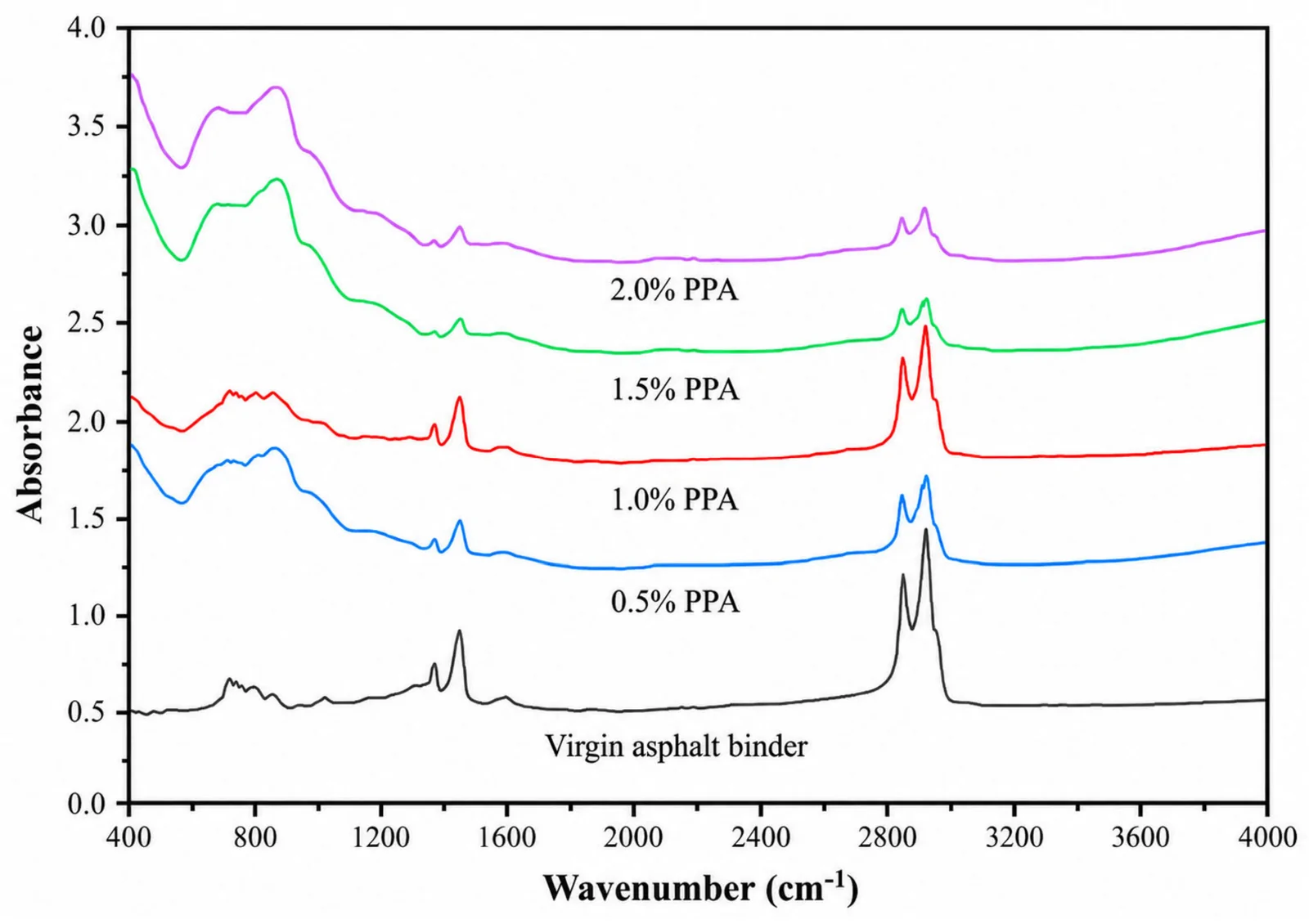
FTIR spectra of virgin and PPA-modified bitumen.

The FTIR spectra of the base asphalt and PPA-modified binders (0.5–2.0%) show the characteristic absorption bands associated with the hydrocarbon structure of asphalt as shown in Figure 13. The bands around 2920 and 2850 cm^−1^ correspond to the asymmetric and symmetric C–H stretching of aliphatic –CH_2_–CH_3_ groups, while the bands near 1455 and 1375 cm^−1^ are associated with CH_2_ and CH_3_ bending vibrations [35,36]. These characteristic bands are retained after PPA modification, indicating that the fundamental hydrocarbon structure of the asphalt binder remains largely unchanged. The band near 1600 cm^−1^ is generally related to aromatic C=C stretching, whereas absorption in the carbonyl region around 1700 cm^−1^ may be associated with oxygen-containing functional groups. Similar characteristic FTIR features have been reported for asphalt and PPA-modified asphalt binders [24,32]. The most noticeable changes occur in the fingerprint region with increasing PPA content from 0.5 to 2.0%, particularly within approximately 1000–1300 cm^−1^. The progressive changes in absorption intensity and the development of additional bands in this region can be associated with phosphate-containing functional groups, including P–O and P=O vibrations, indicating interactions between PPA and polar constituents of the asphalt binder. Previous studies have reported similar spectral changes in PPA-modified asphalt and attributed them to the formation or alteration of phosphate-containing chemical environments within the binder [37]. Therefore, the FTIR results suggest that increasing PPA dosage promotes PPA–asphalt interaction, while the retention of the major hydrocarbon bands indicates that the modification does not fundamentally alter the overall hydrocarbon framework of the asphalt. The gradual increase in these spectral changes with PPA dosage suggests an increasing degree of interaction between PPA and the asphalt binder.

The changes observed in the spectral analysis indicate clearly that the addition of PPA interacts with bitumen chemical composition and specifically with the related region of the phosphate. The changes in the FTIR spectral analysis seem very consistent with the outcomes of the physical and rheological properties of the PPA-modified bitumens in unaged and aged conditions. Similar results in previous studies have related the PPA effect to interactions with the polar component and these can cause the changes in the distribution of the asphaltene which ultimately affect the colloidal structure of the binder. Hence, the spectral analysis in connection with the physical and rheological outcomes directly supports the presence of the physicochemical interaction following the PPA modification.

Recent molecular level study has exhibited that ageing caused due to oxidation involves a succession of dehydrogenation, oxidation and crosslinking reactions, which gradually varies its internal molecular structure [38,39]. These outcomes provide a basis to understand the molecular chemical changes that usually occur during the ageing of bitumen. The changes perceived after RTFO ageing in the phosphate-related FTIR region mainly demonstrate variations in the chemical environment linked with the PPA. Hence, the interaction of the PPA is the bitumen polar component and its presence may affect the ageing due to change in the chemical environment. Conversely, the specific molecular ageing reaction caused by the PPA cannot be assessed from the FTIR results alone.

### 3.5. Limitations of the Study

The outcomes of the present study should be assessed within the scope of the scheme of the programme. First, the study incorporated the only source of bitumen of grade 60/70. Thus, the PPA response may vary with the chemical composition and the original source of the bitumen. Moreover, the bitumen ageing evaluation is limited to only short-term ageing under RTFO conditions. Thus, the response of the ageing does not represent extended long-term ageing. The PAV ageing condition would support a greater understanding of the PPA-modified bitumen’s durability and viscoelastic behaviour. The FTIR analysis was used for the physicochemical interaction while incorporating PPA into the bitumen. The SARA analysis was not evaluated for the direct individual changes within the bitumen fractions though the FTIR outcomes may be considered the evidence of any chemical reaction instead of the direct variations in the colloidal structure of the bitumen. Furthermore, Bending Beam Rheometer (BBR) analysis or Frass temperature were not incorporated in the present study for the low temperature evaluations or any other method. As a result, the PPA addition of 1.0% has shown promising results for balanced performance within the physical and high temperature rheological analysis under unaged, aged, and short-term aged conditions. Conclusively, the morphological analysis, i.e., FESEM, was only utilised for the qualitative surface comparison of a few selected virgin and modified bitumen samples instead of a complete statistical image analysis.

Several practical insights can be acquired from the outcomes of the present study from an engineering perspective. The incorporation of the percentage of PPA that gave the most balanced response when comparing all percentages of PPA may serve as a practical reference for modified PPA bitumens. The preparation of modified bitumen requires the gradual incorporation of PPA, with controlled temperature and adequate mixing for uniform modification. The preparation of modified PPA bitumen at 150 °C with continuous mixing for 20 min may serve as a reference, whereas the other conditions should be further verified in accordance with the virgin bitumen and production system. Furthermore, prolonged higher temperatures during the processing and storage stages should be avoided to prevent the further ageing of, and any possible property changes in, the modified PPA bitumens.

## 4. Conclusions

This study demonstrated that PPA significantly influences the physical, rheological, ageing, and microstructural characteristics of bitumen. Based on the experimental outcomes, the following conclusions can be drawn:

1. Increasing PPA concentration progressively reduced penetration from 62 to 24 dmm and increased the softening point from approximately 46.5 °C to 51, 56, 64, and 68 °C. Similarly, viscosity at 135 °C increased from 0.38 to 0.42, 1.21, 1.62, and 1.82 Pa·s with increasing PPA dosage, although all binders remained below the 3 Pa·s limit.
2. The PI and PVN results confirmed a considerable reduction in temperature susceptibility, while the ageing indices showed that the effect of PPA on short-term ageing varied with concentration, temperature, and the property considered. This indicates that ageing susceptibility cannot be adequately represented by a single conventional parameter.
3. The rheological results showed a pronounced improvement at approximately 1.0% PPA, where G*/sinδ increased from 1.01 to 2.37, 12.35, 11.93, and 20.73 kPa at 0.5%, 1.0%, 1.5%, and 2.0% PPA, respectively, and the high temperature grade increased from PG 64 to PG 82. Further increases in PPA concentration increased stiffness and elastic response but did not produce an additional improvement in the measured performance grade.
4. Master curve analysis showed that the influence of PPA was more pronounced at lower reduced frequencies, while the Black and Cole–Cole diagrams confirmed changes in the viscoelastic response towards a greater elastic contribution. The most pronounced transition in rheological response generally occurred between 0.5% and 1.0% PPA. These characteristics remained evident following RTFO ageing.
5. FESEM showed homogeneous incorporation of 1.0% PPA without visible phase separation, with more pronounced morphological changes following ageing. FTIR further indicated progressive changes in the phosphate-related spectral region while the principal hydrocarbon structure remained largely unchanged.
6. Considering the combined physical, rheological, ageing, and microstructural results, 1.0% PPA provided the most promising overall balance among the investigated concentrations, whereas higher concentrations mainly resulted in additional stiffening without proportional improvement across the evaluated characteristics.

## Figures and Tables

**Table 1:** Basic properties of virgin bitumen and PPA.

Material	Property	Value	Standard
Bitumen	Penetration at 25 °C	62	ASTM D5
	Softening point	46.4	ASTM D36
	Viscosity at 135 °C & 165 °C (Pa·s)	0.377 & 0.108	ASTM D4402
	Specific gravity	1.030	ASTM D70
	Ductility @ 10 °C (cm)	118	ASTM D113
	Mass loss (%)	1.12	ASTM D2872
PPA	Physical form	Viscous liquid	
	Appearance	Colourless	
	H_3_PO_4_ equivalent concentration	115%	
	Specific gravity	2.06	
	Viscosity at 25 °C	1.10 Pa·s	
	Boiling point	300 °C	
	Specific heat capacity	1.27 J/(g °C)	

**Table 2:** Basic properties of virgin bitumen and PPA.

Property	Condition	0% PPA	0.5% PPA	1.0% PPA	1.5% PPA	2.0% PPA	ANOVA *p* Value
Penetration (dmm)	Unaged	62.07 ± 1.10 ^a^	57.33 ± 0.64 ^b^	48.20 ± 0.92 ^c^	39.07 ± 1.42 ^d^	24.53 ± 0.42 ^e^	<0.001
Penetration (dmm)	RTFO	48.60 ± 0.72 ^a^	48.93 ± 0.90 ^a^	38.40 ± 0.80 ^b^	20.00 ± 1.22 ^c^	17.20 ± 1.00 ^d^	<0.001
Softening point (°C)	Unaged	46.47 ± 0.65 ^a^	54.13 ± 0.10 ^b^	56.13 ± 0.67 ^c^	63.93 ± 0.37 ^d^	67.88 ± 0.64 ^e^	<0.001
Softening point (°C)	RTFO	50.03 ± 0.18 ^a^	54.17 ± 0.71 ^b^	64.13 ± 0.40 ^c^	69.00 ± 0.79 ^d^	73.52 ± 0.55 ^e^	<0.001
Viscosity at 135 °C (Pa·s)	Unaged	0.378 ± 0.014 ^a^	0.422 ± 0.003 ^a^	1.212 ± 0.012 ^b^	1.615 ± 0.075 ^c^	1.823 ± 0.072 ^d^	<0.001
Viscosity at 135 °C (Pa·s)	RTFO	0.694 ± 0.015 ^a^	0.645 ± 0.099 ^a^	1.801 ± 0.013 ^b^	2.142 ± 0.262 ^c^	2.714 ± 0.031 ^d^	<0.001
Viscosity at 165 °C (Pa·s)	Unaged	0.108 ± 0.008 ^a^	0.123 ± 0.001 ^a^	0.400 ± 0.024 ^b^	0.496 ± 0.039 ^c^	0.681 ± 0.015 ^d^	<0.001
Viscosity at 165 °C (Pa·s)	RTFO	0.142 ± 0.008 ^a^	0.400 ± 0.041 ^b^	0.485 ± 0.041 ^b^	0.713 ± 0.040 ^c^	1.125 ± 0.071 ^d^	<0.001

Values are mean ± standard deviation. Superscript letters indicate Tukey’s HSD groups within each row. Values sharing the same letter are not significantly different, while different letters indicate significant differences at *p* < 0.05.

**Table 3:** PI, PVN, and ageing indices of PPA-modified bitumens.

Binder	Penetration Index (PI)	Penetration Viscosity Number (PVN)	Penetration Ageing Index (PAI)	Softening Ageing Index (SAI)	Viscosity Ageing Index (VAI)	
					**135 °C**	**165 °C**
Virgin	−1.64	−0.79	0.78	1.08	1.84	1.31
0.5	−0.61	−0.71	0.86	1.06	1.53	3.26
1.0	0.13	0.46	0.79	1.14	1.49	1.21
1.5	1.18	0.58	0.51	1.08	1.33	1.46
2.0	0.86	0.28	0.69	1.08	1.49	1.64

**Table 4:** Temperature failures and performance grading.

Binder	G*/sin (δ) (kPa)	δ (°)	Failure Temperature	PG Grade
Virgin	1.01	88.70	64.2	64
0.5	2.37	81.0	72.40	70
1.0	12.35	61	>82	82
1.5	11.93	61.70	>82	82
2.0	20.73	55.10	>82	82
Virgin-aged	2.08	87.30	64	64
0.5	4.79	76.30	69	70
1.0	9.92	63.40	>82	82
1.5	9.92	62.90	>82	82
2.0	19.36	53.20	>82	82

## Data Availability

The original contributions presented in this study are included in the article. Further inquiries can be directed to the corresponding authors.

## Abbreviations and Symbols

**Abbreviation**	**Definition**	
DSR	Dynamic shear rheometer	
FESEM	Field emission scanning electron microscopy	
FTIR	Fourier transform infrared spectroscopy	
PAI	Penetration ageing index	
PG	Performance grade	
PI	Penetration index	
PPA	Polyphosphoric acid	
PVN	Penetration viscosity number	
RTFO	Rolling thin film oven	
SAI	Softening point ageing index	
VAI	Viscosity ageing index	
WLF	Williams Landel Ferry	
**Symbol**	**Definition**	**Unit**
aT	Shift factor	—
C1, C2	WLF model constants	—
fr	Reduced frequency	Hz
G*	Complex shear modulus	Pa or kPa
G′	Elastic modulus	Pa or kPa
G″	Viscous modulus	Pa or kPa
G*/sinδ	Rutting parameter	kPa
T	Testing temperature	°C
Tref	Reference temperature	°C
δ	Phase angle	°
